# Use of the mCherry fluorescent protein to optimize the expression of class I lanthipeptides in *Escherichia coli*

**DOI:** 10.1186/s12934-023-02162-7

**Published:** 2023-08-09

**Authors:** Winschau F Van Zyl, Anton D. Van Staden, Leon M T. Dicks, Marla Trindade

**Affiliations:** 1 Department of Microbiology, Stellenbosch University, Cape Town, South Africa; 2 Institute for Microbial Biotechnology and Metagenomics, University of the Western Cape, Cape Town, South Africa; 3 Division of Clinical Pharmacology, Department of Medicine, Faculty of Medicine and Health Sciences, Stellenbosch University, Cape Town, South Africa; 4 Department of Microbiology and Biochemistry, University of the Free State, Bloemfontein, South Africa

**Keywords:** Class I lanthipeptides, *E. coli*, Heterologous expression, mCherry-fusion, Real-time in vivo monitoring, Optimization of expression

## Abstract

**Background:**

Lanthipeptides are a rapidly expanding family of ribosomally synthesized and post-translationally modified natural compounds with diverse biological functions. Lanthipeptide structural and biosynthetic genes can readily be identified in genomic datasets, which provides a substantial repository for unique peptides with a wide range of potentially novel bioactivities. To realize this potential efficiently optimized heterologous production systems are required. However, only a few class I lanthipeptides have been successfully expressed using *Escherichia coli* as heterologous producer. This may be attributed to difficulties experienced in the co-expression of structural genes and multiple processing genes as well as complex optimization experiments.

**Results:**

Here, an optimized modular plasmid system is presented for the complete biosynthesis for each of the class I lanthipeptides nisin and clausin, in *E. coli.* Genes encoding precursor lanthipeptides were fused to the gene encoding the mCherry red fluorescent protein and co-expressed along with the required synthetases from the respective operons. Antimicrobially active nisin and clausin were proteolytically liberated from the expressed mCherry fusions. The mCherry-NisA expression system combined with in vivo fluorescence monitoring was used to elucidate the effect of culture media composition, promoter arrangement, and culture conditions including choice of growth media and inducer agents on the heterologous expression of the class I lanthipeptides. To evaluate the promiscuity of the clausin biosynthetic enzymes, the optimized clausin expression system was used for the heterologous expression of epidermin.

**Conclusion:**

We succeeded in developing novel mCherry-fusion based plug and play heterologous expression systems to produce two different subgroups of class I lanthipeptides. Fully modified Pre-NisA, Pre-ClausA and Pre-EpiA fused to the mCherry fluorescence gene was purified from the Gram-negative host *E. coli* BL21 (DE3). Our study demonstrates the potential of using in vivo fluorescence as a platform to evaluate the expression of mCherry-fused lanthipeptides in *E. coli*. This allowed a substantial reduction in optimization time, since expression could be monitored in real-time, without the need for extensive and laborious purification steps or the use of in vitro activity assays. The optimized heterologous expression systems developed in this study may be employed in future studies for the scalable expression of novel NisA derivatives, or novel genome mined derivatives of ClausA and other class I lanthipeptides in *E. coli*.

**Supplementary Information:**

The online version contains supplementary material available at 10.1186/s12934-023-02162-7.

## Background

Lanthipeptides are ribosomally synthesized and post-translationally modified to produce lanthionine and methyl lanthionine ring structures. Currently, they are grouped into five classes based on amino acid (AA) sequences of unmodified core peptides and the enzymes involved in modifications [1, 2]. Precursor lanthipeptides (LanA) modifications are introduced by either a dedicated dehydratase (LanB) and a cyclase (LanC), for class I lanthipeptides, or a single bifunctional synthetase (LanM) that catalyses dehydration and cyclization, as described for class II lanthipeptides [3]. Serine and threonine residues are dehydrated to form 2,3-didehydroalanine (Dha) and 2,3-didehydrobutyrine (Dhb), respectively [4, 5]. The nucleophilic addition of Cys residues to Dha and Dhb, introduced by cyclase LanC, results in the formation of lanthionine and 3-methyl-lanthionine, respectively [6–8]. The thioether cross-links render rigidity and stability to the peptides [9]. Further modifications may include oxidative decarboxylation of the C-terminus, formation of a lactate group at the N-terminus, and introduction of D-amino acids [10–12]. All lanthipeptides have ribosomal-synthesized precursor peptides composed of a N-terminal leader peptide (LP) acting as a docking station for modification enzymes, and a C-terminal core peptide (CP) that undergoes modifications [13]. Lanthipeptides are produced as pre-lanthipeptides (precursor peptides with modification on the core peptide attached to the N-terminal LP) to protect the expression host. These precursor peptides become active as soon as the LP is proteolytically removed by a dedicated lanthipeptide protease (LanP).

Nisin is the best-studied and only lantibiotic (lanthipeptide with antimicrobial activity) approved to use as a food preservative [6, 14, 15]. The discovery that some lanthipeptides have antiviral-, anticancer and analgesic properties, modulate immune responses, and regulate the functioning of ion channels, shifted the attention to medical applications [1]. Many of these peptides are, however, unstable when used in vivo [14, 16–18]. Chemical synthesis of lanthipeptides is expensive and is thus not a viable option [19]. Lanthipeptides have been successfully produced in Gram-positive hosts, including *Lactococcus lactis*, *Bacillus* spp., *Streptomyces lividans* and *Corynebacterium glutamicum* [1, 20–24]. Genetic manipulation and the expression of heterologous genes encoding lanthipeptides and synthetases in wild-type producing hosts proved effective, but only for some lanthipeptides [25–27]. As the native producer of nisin, *L. lactis* has been employed as a suitable host for the expression of class I and class II peptides using the required modification enzymes from each class [1]. Apart from low production yields, wild-type strains may become sensitive to the peptides once post-translationally modified. The answer to the problem may be the design of robust lanthipeptide expression systems optimised for *Escherichia coli*. Several lanthipeptide genes have been expressed by *E. coli* [1, 18]. The biggest advantage is that *E. coli* grows rapidly on inexpensive media with a variety of strains, cloning tools and expression systems available [1, 20]. Moreover, *E. coli* may not require all genes in the biosynthesis cluster, as reported for several lanthipeptides from all four classes [1]. The omission of non-essential genes, like those involved in host immunity and regulation, may limit the metabolic burden on *E. coli* and increase production levels. Nisin was the first lanthipeptide expressed by *E. coli* [28]. The authors used a compatible dual vector approach to co-express the nisin precursor peptide, dehydratase (NisB) and cyclase (NisC). This paved the way for the biosynthesis and modification of lanthipeptides from all four classes [1]*.* However, only a few class I lanthipeptides have been successfully expressed in *E. coli*. The functional expression of lanthipeptides may be complicated by many unpredictable factors, such as the co-expression of core peptide and multiple processing genes, promoter strength, basal level expression, product toxicity, host strain selection, plasmid copy number, inducer agent and composition of growth medium [20, 29, 30]. A careful balance of promoter strength and control is necessary for the optimized expression of all bacteriocins [20, 29]. Moreover, to minimize the metabolic burden on the host prior to the induction phase, promoter strength is a critical factor since un-induced overexpression of lanthipeptides and their much larger post-translational modification enzymes can be harmful to the normal functioning of the host cells. Thus, the choice of promoter must be strong and have an effective induction system to prevent background basal level expression that may place a burden on the host resources before the production phase. Furthermore, inducible protein expression systems usually require complex optimization steps, such as accurate control of growth temperature for expression, choosing the correct inducer and promoter, and optimal growth media. Based on the relatively small size of lanthipeptides (2–7 kDa), their overexpression may have little physiological effect on the host. On the other hand, overexpression of the much larger lanthipeptide post-translational modification (PTM) enzymes (i.e. ± 117 kDa for NisB) may require more extensive resources for its biosynthesis and therefore require controlled expression using inducible promoters for expression. Such a strategy is reliant on the tight control of the inducible promoter (i.e., be highly repressible) since the expression of modification enzymes may require a slower process from transcription to translation for proper folding and solubilisation of the proteins. Precursor lanthipeptides may also be susceptible to proteolytic degradation and lantibiotics such as nisin and epidermin show limited stability and low solubility at neutral pH compared to the more stable globular peptides such as mersacidin [31–33]. The class I LanB dehydratases are also aminoacyl-tRNA dependent and may require the inclusion of optimized tRNAGlu sequences and glutamyl-tRNA synthetase to increase the efficiency of class I modification enzymes [34, 35]. Another major limiting factor is the sequestration of heterologously produced peptides to inclusion bodies, limiting the access of precursor peptides to modification enzymes in the cytosol [36]. A potential solution to the latter is the fusion of precursor peptides to larger fusion partners that enhances solubility, reduces toxicity and increases contact time between the fusion protein and synthetases in the cytosol [37]. Fusion of a large, soluble, protein to the N-terminus of the LP may reduce the toxicity of precursor peptides, without interfering with peptide modifications [38]. Examples are the expression of the lanthipeptides haloduracin β fused to mannose-binding protein, and ruminococcin A and nisin A fused to green fluorescent protein (GFP) [37–39].

In this study, we utilized the Duet™ expression vectors to construct an optimized plug-and-play system for the complete biosynthesis and purification of each of the lanthipeptides nisin and clausin, in *E. coli.* Co-expression of LanA, LanB, LanC and LanD was achieved using a plasmid system with compatible replication origins and different antibiotic selection markers for maintenance of plasmids in *E. coli.* Each system consists of a minimal gene cluster, expressing three (*nisABC*) of the 11 genes present in the nisin operon and four (*clausABCD*) of the 9 genes present in the clausin operon [6, 33]. Sequence and structural similarities of the core peptides and PTM enzymes classify nisin (nisin-like) and clausin (epidermin-like) as class I lantibiotics [1]. Based on these similarities, the optimized expression conditions for NisA were directly applied to produce Clausin. Furthermore, as proof of principle, the optimized clausin expression system was used to evaluate the heterologous expression of epidermin.

Genes encoding precursor lanthipeptides were fused to the gene encoding the red fluorescent protein mCherry. We show that in vivo fluorescence imaging can be successfully used as a tool to estimate the impact of key factors that can affect the production of class I lanthipeptides in *E. coli*. The effect of different culture conditions including growth media composition; the use of glucose in pre-growth media to control basal expression levels; and glycerol and IPTG (thio-β-d-galactopyranoside) as inducer agents was determined using real-time in vivo fluorescence monitoring of NisA expression. In addition, the influence of promoter arrangement on the heterologous synthesis of lanthipeptides, which require the co-expression of multiple genes, was studied. Precursor lanthipeptides were overexpressed using the strong STldh promoter, previously shown to be one of the strongest promoters directing high level expression in *Lc. lactis*, *Lactobacillus plantarum*, *Listeria monocytogenes* and *E. coli* [40–42]. The optimized heterologous bioprocesses developed in this study may be employed in future studies as a useful tool for the optimized production of diverse class I lanthipeptides in *E. coli*.

## Results

### Influence of promoter on driving the co-expression of mCherry-NisA and NisB in pRSTldhCherry

**Fig. 1:**
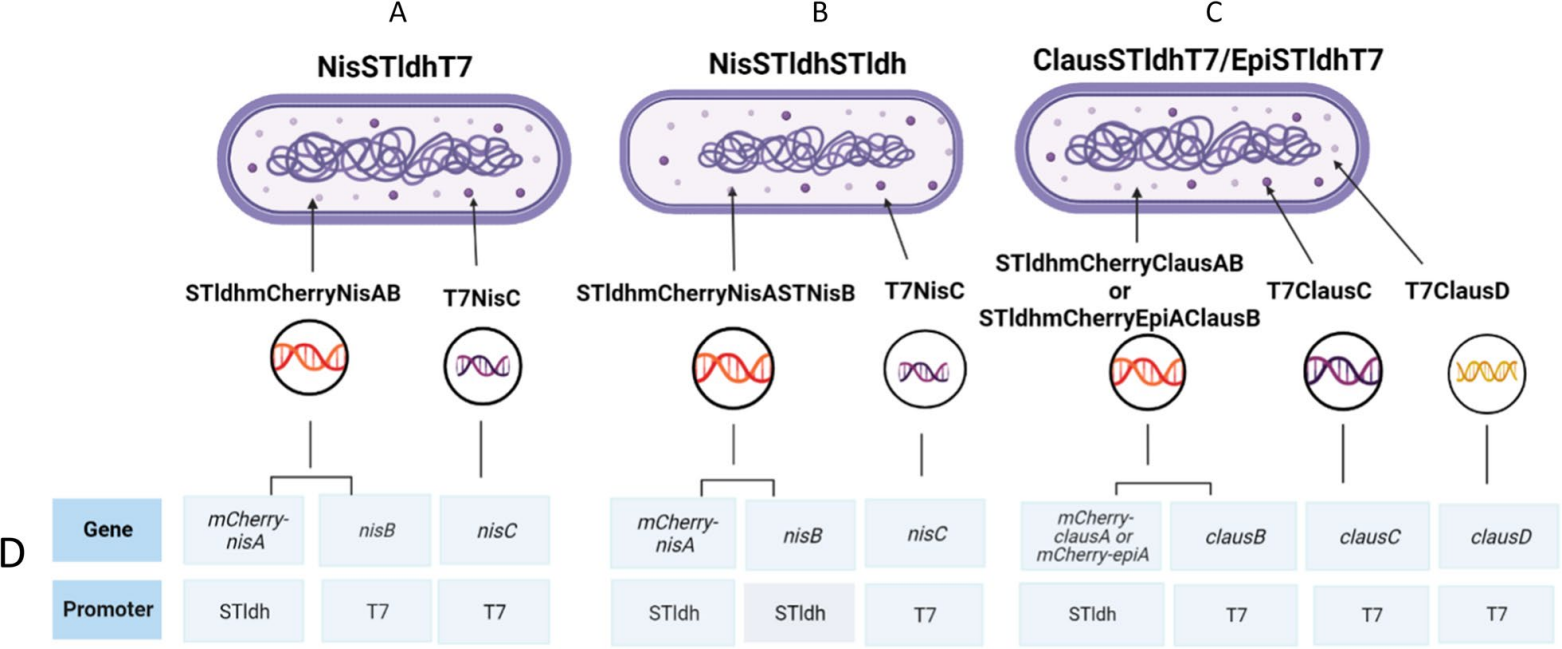
Overview of lanthipeptide expression strains constructed in this study. **A** The NisSTldhT7 mCherry-NisA heterologous expression strain was co-transformed with plasmids pRSTcnA*nB and pACYCNisCi. **B** Strain NisSTldhSTldh was co-transformed with pRSScnA*nB and pACYCNisCi. The Pre-ClausA and Pre-EpiA-producing strains were co-transformed with pRSTccA*cB (for clausin) or pRSTceA*cB (for epidermin) and plasmids pClausCi and pClausDi to yield strains **C** ClausSTldhT7 and EpiSTldhT7, respectively. Relevant promoter and gene combinations are indicated in **D**. Figure created in biorender (http://biorender.io)

**Fig. 2:**
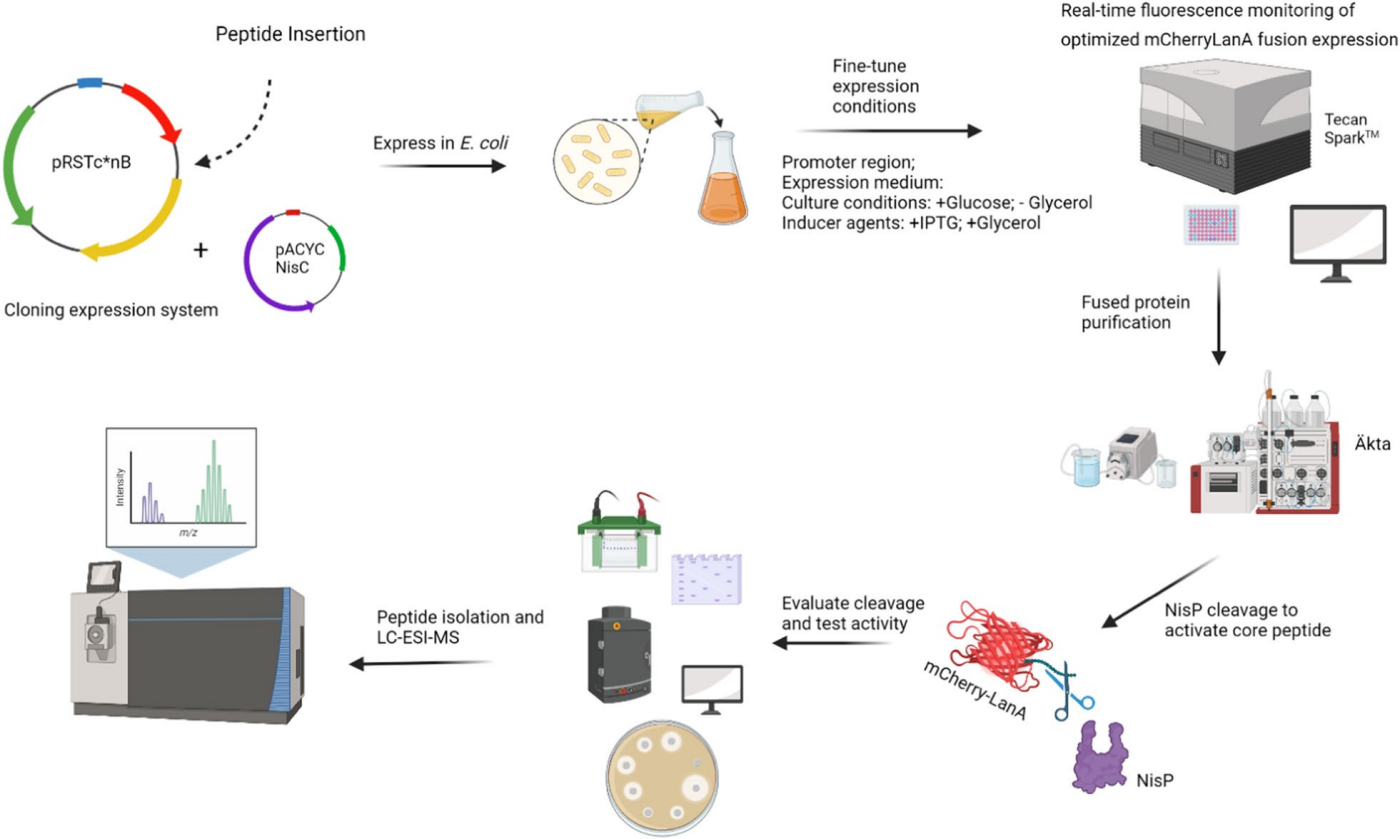
Overall strategy used to produce, activate and purify class I lanthipeptides in *E. coli.* Figure created in biorender (http://biorender.io)

Pre-testing the effect of different promoter arrangements when using the STldh promoter for the expression of mCherry-nisA (MCS1) and NisB (MCS2**)** (strain NisSTldhSTldh, Fig. 1B) compared to the use of STldh for expression of mCherry-nisA (MCS1) and the T7 promoter for NisB (MCS2) (strain NisSTldhT7, Fig. 1A) was accomplished by monitoring in vivo fluorescence signals of *E. coli* BL21 carrying the respective constructs. The overall strategy for lanthipeptide production, activation, and purification is illustrated in Fig. 2.

**Fig. 3:**
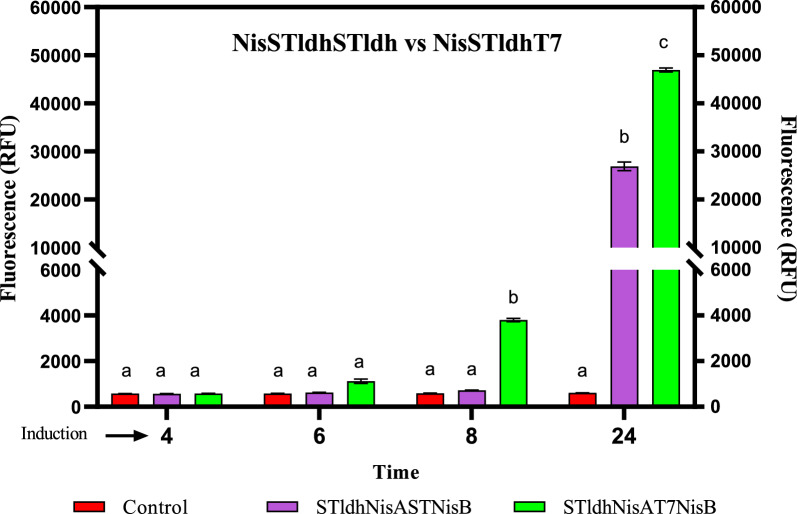
Comparison of fluorometric intensity measurements of *E. coli* BL21 strains NisSTldhSTldh and NisSTldhT7, expressing mCherry-NisA cultured in terrific broth containing glucose (1% v/v) and induced with 0.15 mM IPTG and 0.5% glycerol (v/v). STldhSTldh: STldh promoter used for expression of mCherry-NisA and NisB; STldhT7: STldh used for expression of mCherry-NisA and T7 promoter for NisB. Means (SEM) from three independent (n = 3) cultures are shown. Different single-letter combinations indicate which groups have significance at different time points and should be interpreted as (a) vs (a) = no difference; (a) vs (b) = different and (b) vs (c) = different. Error bars show standard deviation. Statistical differences were determined by two-way ANOVA (*P* < 0.05, *P* < 0.01 and* P* < 0.001)

Based on fluorescence output, decreased levels of expression were detected when the STldh promoter was used to drive expression in both MCS1 and MCS2 (Fig. 3). Although both combinations of promoters were feasible for mCherry-NisA expression, the use of STldh in MCS1 and T7 in MCS2 resulted in the highest expression (twofold) after 24 h (Fig. 3). Results in Additional file 1: Fig. S1 show that both strains shared similar growth characteristics.

**Fig. 4:**
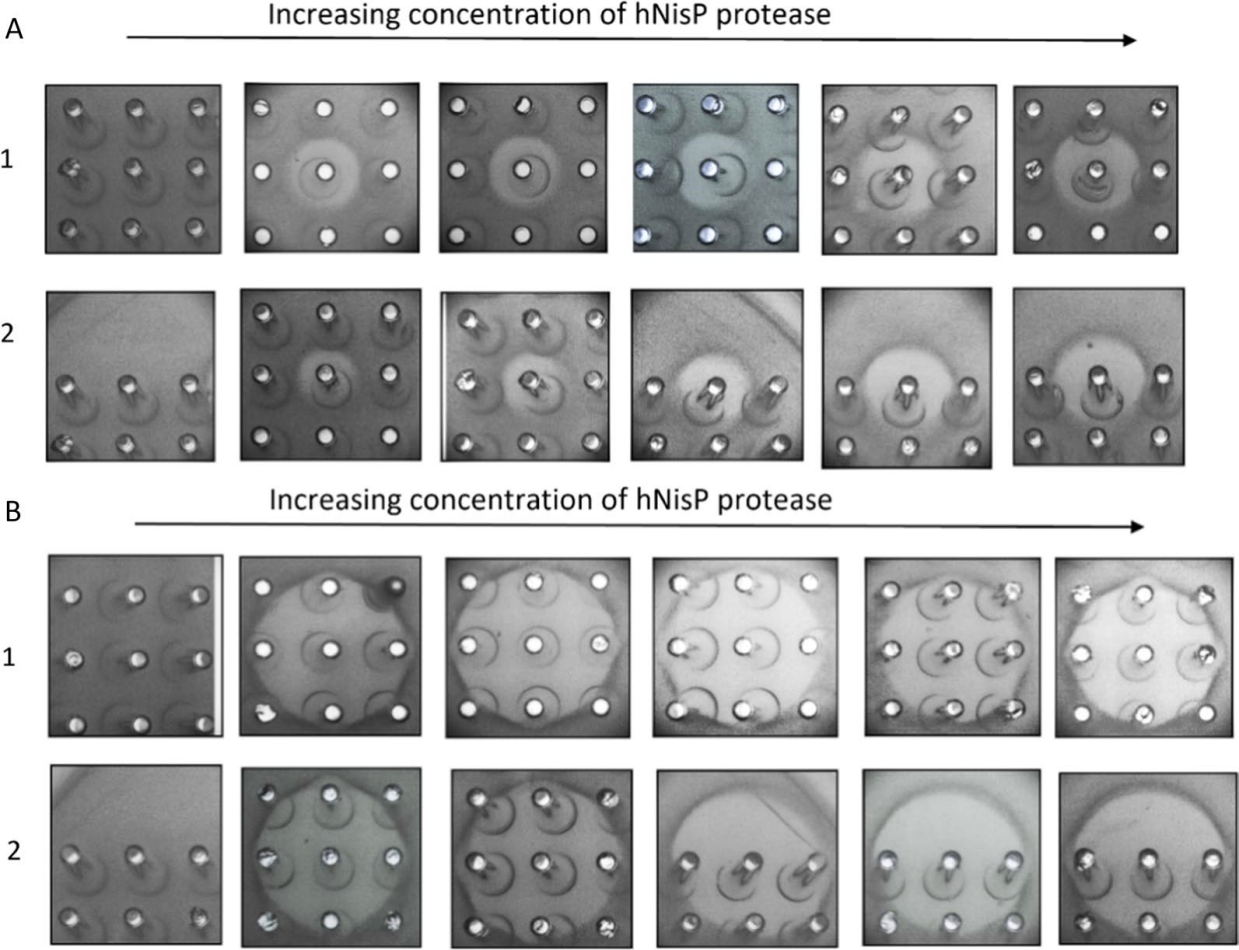
Antimicrobial activity of mCherry-NisA at various hNisP cleavage concentrations. Antimicrobial activity of **A** mCherry-NisA purified from strain NisSTldhSTldh and **B** mCherry-NisA purified from stain NisSTldhT7. mCherry-NisA cleavage was performed at 26 °C for (**1**) 4 h and (**2**) 20 h, respectively. Cleavage was assessed using a spot plate assay against *L. sakei*. hNisP was added at concentrations 0, 0.01, 1.1, 15, 120, and 300 ng/µL. Post cleavage, 50 µL of mCherry-NisA was spotted from each cleavage reaction

mCherry-NisA produced by *E. coli* BL21 carrying constructs with each respective promoter combination were purified by immobilized metal affinity chromatography (IMAC), desalted and cleaved with hNisP (Fig. 2) at an increasing range of concentrations to compare levels of inhibition based on cleavage activation (Fig. 4).

All antimicrobial activity assays were standardized in terms of fused-peptide and hNisP concentrations and total reaction volumes to account for possible differences in PTM enzyme activities. Cleavage resulted in the liberation of active peptide at all concentrations tested, after 4 or 24 h of cleavage. No antimicrobial activity was detected for uncleaved samples (Fig. 4A, B). Most notably, a decrease in activity was observed when expression was performed using STldh in both MCSs (Fig. 4) and is consistent with the significantly lower fluorescence levels observed for these constructs (Fig. 3). By using the respective promoter arrangements, an mCherry-NisA yield of 15 mg was obtained with strain NisSTldhSTldh and 24 mg with strain NisSTldhT7 (both per liter *E. coli* culture). Considering the size-ratio of mCherry+NisA leader-to-core segments (~ 9:1), these amounts would generate final yields of approximately 1.5 mg (strain NisSTldhSTldh) and 2.4 mg (strain NisSTldhT7) of pure and active nisin per liter of culture when the mCherry protein and leader peptide are removed using hNisP (120 ng/µL for 24 h). In addition, highest mCherry-PreNisA production in terms of visual brightness can be clearly identified in the expression media after 24 h incubation when using STldh in MCS1 and T7 in MCS2 (Additional file 1: Fig. S10). Based on these results, the NisSTldhT7 strain was used to carry out the optimization experiments described in this study.

### Fluorometric optimization of mCherry-NisA expression levels under different culture conditions

**Fig. 5:**
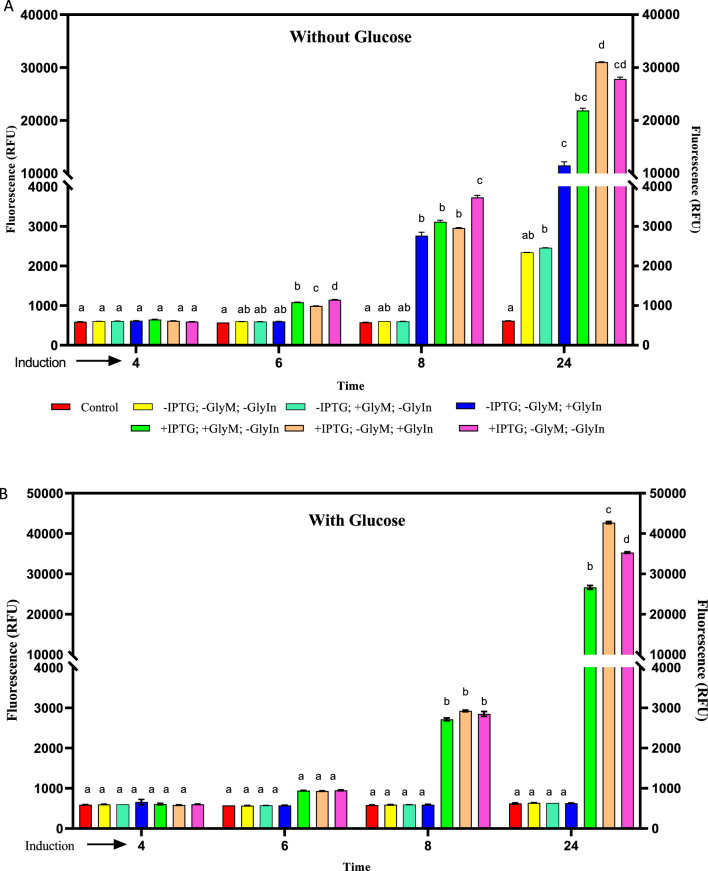
Fluorometric measurements of strain NisSTldhT7 expressing mCherry-NisA under various culturing and induction conditions. Cultures were grown in terrific broth supplemented without (**A**) and with (**B**) glucose (1% v/v) for 24 h. Culturing and induction conditions included: (−IPTG; −GlyM; −GlyIn) no induction; (−IPTG; +GlyM; −GlyIn) no IPTG induction with glycerol (0.5% v/v) added to pre-growth media; (−IPTG; −GlyM; +GlyIn) no IPTG induction but induced with glycerol (0.5% v/v); (+IPTG; +GlyM; −GlyIn) induced with 0.15 mM IPTG and glycerol added to pre-growth media; (+IPTG; −GlyM; +GlyIn) induced with 0.15 mM IPTG and glycerol; (+IPTG; −GlyM; −GlyIn) induced with 0.15 mM IPTG. Fluorescence intensity was measured in relative fluorescence units (RFU Log_10_). Means (SEM) from three independent (n = 3) cultures are shown with standard deviations indicated by error bars. Different single or double-letter combinations indicate which groups have significance at different time points and should be interpreted as (a) vs (a) = no difference; (a) vs (b) = different; (b) vs (c) = different and (ab) vs (bc) = different. Statistical differences were determined by two-way ANOVA (*P* < 0.05, *P* < 0.01, and* P* < 0.001). *IPTG* Thio-B-d-galactopyranoside, *GlyM* glycerol presence in pre-growth media, *GlyIn* glycerol used as inducer agent; +: added and −: not added

Glucose is easily metabolized by cells and thus an ideal substrate for cell growth in *E. coli* during the induction phase, prior to the addition of IPTG to the medium. Glycerol is a heavier carbon source compared to glucose and its metabolism causes cyclic AMP (cAMP) levels to rise for enhanced expression from promoters controlled by catabolite repression. Consequently, the separate and combined effects of glucose, glycerol, and IPTG addition to expression media, or their inclusion in expression media, as inducer agents on the heterologous expression of the mCherry-NisA fusion protein were monitored in vivo and in real-time. Freshly co-transformed isolates of strain NisSTldhT7 (Fig. 1A) were used for fluorescence monitoring experiments. The fluorometric output of the culture group grown in the absence of glucose or glycerol, and without IPTG or glycerol induction (at OD600nm of 0.6) emitted a minimal increase in fluorescence compared to the non-fluorescent control strain after 8 h and increased fourfold after 24 h (Fig. 5A, −IPTG; −GlyM; −GlyIn). In the same group, no fluorescence was detected over the 24 h incubation period when cells were cultured in the presence of 1% glucose (Fig. 5B, −IPTG; −GlyM; −GlyIn). Similarly, no fluorescence was observed when cultures were grown in the combined presence of glucose and 0.5% glycerol in pre-growth media (Fig. 5B; −IPTG; +GlyM; −GlyIn) or when only glycerol was used for induction (Fig. 5B, −IPTG; −GlyM; +GlyIn). In contrast, the fluorometric output of cultures induced with glycerol alone and grown without glucose supplementation (Fig. 5A, −IPTG; −GlyM; +GlyIn) increased significantly after 8 h incubation compared to cultures grown in media pre-supplemented with 0.5% glycerol (Fig. 5A, −IPTG; +GlyM; −GlyIn). After 24 h, an additional fivefold increase in fluorescence was detected when glycerol was used as inducer agent (Fig. 5A, −IPTG; −GlyM; +GlyIn) on its own compared to the un-induced culture group (Fig. 5A, −IPTG; −GlyM; −GlyIn) or when TBG (TB containing 0.5% glycerol), was used as expression medium (Fig. 5A, −IPTG; +GlyM; −GlyIn). Induction with IPTG at OD_600nm_ of 0.6 resulted in significantly higher fluorescence emission with or without glucose supplementation of the pre-growth media after 24 h incubation (Fig. 5A, B). Maximal fluorescence emission was recorded after 24 h when cultures were grown in media supplemented with glucose only and induced with IPTG and glycerol during exponential growth (Fig. 5B, +IPTG; −GlyM; +GlyIn). In relation to the fluorescence output, the cell density of strain NisSTldhT7 increased over the entire cultivation period and shared similar growth characteristics for all culturing and inducing conditions (Additional file 1: Fig. S2A, B).

### Optimization of mCherry-NisA expression in different culture media

**Fig. 6:**
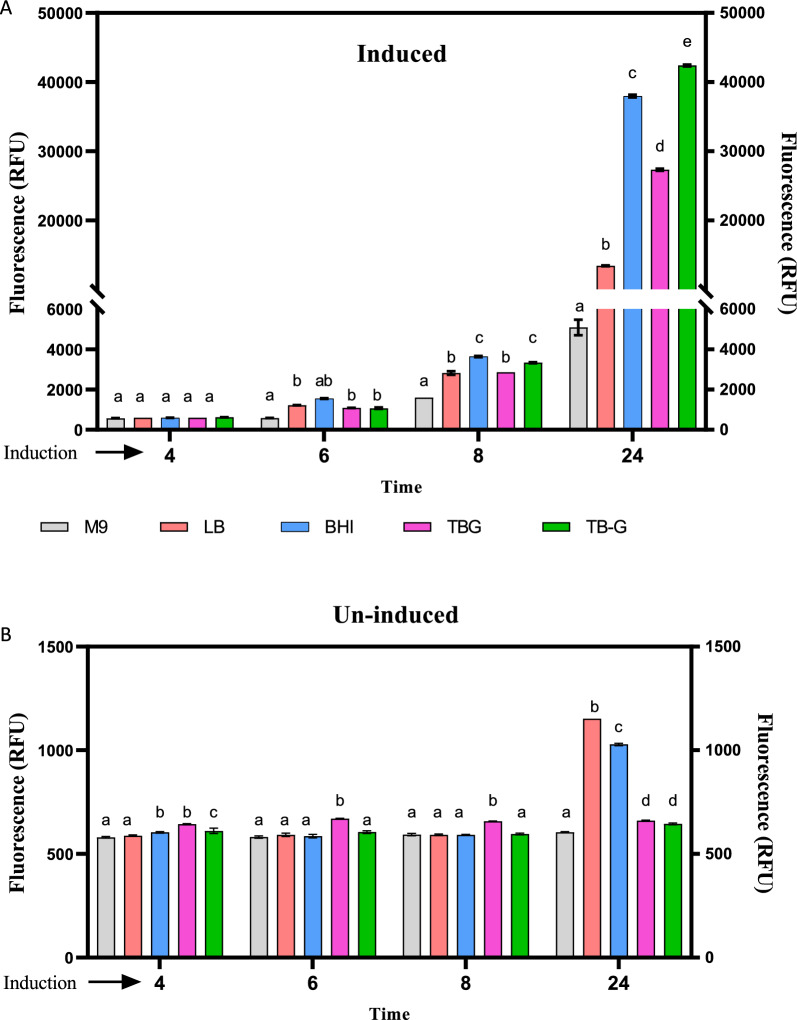
Fluorometric intensity of *E. coli* BL21 expressing mCherry-NisA in M9 minimal media, LB, BHI, TBG (TB containing 0.5% glycerol), TB-G (TB without 0.5% glycerol). **A** In vivo fluorometric measurements of cultures induced with 0.15 mM IPTG and 0.5% glycerol (v/v). **B** In vivo fluorometric measurements of un-induced cultures. Means (SEM) from three independent (n = 3) cultures are shown with standard deviations indicated by error bars. Statistical differences were determined by two-way ANOVA (*P* < 0.05, *P* < 0.01 and* P* < 0.001). Different single-letter combinations indicate which groups have significance at different time points and should be interpreted as (a) vs (a) = no difference; (a) vs (b) = different; (b) vs (c) = different and (c) vs (d) different

Pre-selection of the optimal growth media for NisA expression was accomplished by monitoring fluorescence signals emitted by strain NisSTldhT7 (Fig. 1A). Cells were cultivated in LB, BHI, TBG (TB containing 0.5% glycerol), TB-G (TB without 0.5% glycerol) and M9 culture media. All pre-growth media were supplemented with 1% glucose based on results established in Fig. 5. Fluorescence emission of induced cultures [0.15 mM IPTG and 0.5% glycerol (v/v)] increased with time and were significantly affected by the media used for expression (Fig. 6A). The weakest overall fluorescence signals were detected when induced cells were cultured in M9 minimal media after 24 h incubation compared to all other mediums tested (Fig. 6A). No significant difference in fluorescence was observed between cultures grown in BHI or TB-G after an 8-h incubation period (Fig. 6A). After 24 h, highest fluorescence emission was detected when cells were cultured in TB-G (Fig. 6A). Significant differences in fluorescence output between cells grown in TB-G, TGB or BHI were confirmed with a four- and twofold increase after 24 h, respectively (Fig. 6A).

Fluorescence output of un-induced (no IPTG or glycerol added) *E. coli* BL21 cells carrying mCherry-NisA constructs grown in M9, LB, BHI, TBG or TB-G were monitored to determine the levels of background basal expression in each medium (Fig. 6B). Un-induced cells grown in TBG, emitted significantly higher fluorescence after 8 h incubation compared to other mediums tested (Fig. 6B). No significant differences in fluorescence were recorded after 24 h between un-induced cells grown in TBG or TB-G, whereas highest basal level fluorescence emission was recorded when grown in LB and BHI, respectively (Fig. 6B). Additionally, levels of un-induced and induced expression of mCherry-NisA was easily distinguishable by the appearance of white/cream (un-induced) and varying levels of purple (induced) cell pellets after centrifugation (Additional file 1: Fig. S12). The brightest purple pellets were obtained consistently when TB-G and BHI was used as growth media, whereas the use of M9 and TBG yielded a decreased colour change. Growth profiles of induced and un-induced cultures in relation to fluorescence signals is shown in Additional file 1: Fig. S3A, B.

**Fig. 7:**
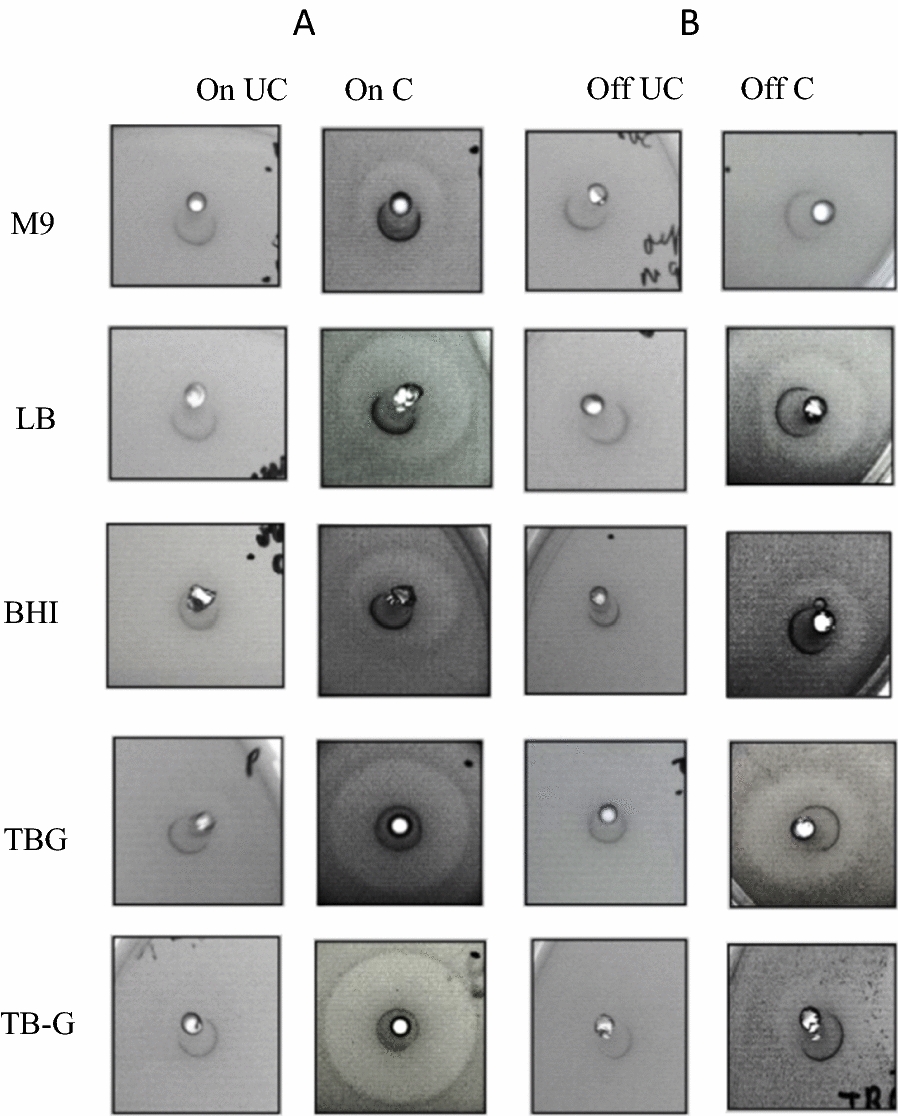
Antimicrobial activity and optimization results for mCherry-PreNisA expressed in M9 minimal media, LB, BHI, TBG (TB containing 0.5% glycerol) and TB-G (TB without 0.5% glycerol). Antimicrobial activity of IMAC-purified mCherry-PreNisA produced by cultures that were **A** induced (On) with 0.15 mM IPTG and 0.5% glycerol (v/v) and those that were kept **B** un-induced (off). Uncleaved (UC) and hNisP cleaved (C) activity results are shown for all samples tested. Antimicrobial activity was assessed using an agar well diffusion assay against *L. sakei*

mCherry-NisA expressed in each of the respective growth mediums (induced and un-induced) were purified by IMAC, desalted, and cleaved with hNisP to determine differences in antimicrobial activity against *L. sakei* (Fig. 7). Using the heterologous expression system optimized in this study, successful expression of mCherry-NisA was confirmed by the detection of clear zones of inhibition (Fig. 7A, On C). No background activity was detected from uncleaved mCherry-NisA, regardless of induction or the media used for expression (Fig. 7A, On UC; and Fig. 7B Off UC). The largest activity zones for hNisP cleaved mCherry-NisA was observed when TB-G was used as expression media (Fig. 7A, On C). These results correlate with the level of activity and fluorescence output observed in Figs. 3, 4, 5A and 6A, respectively, when cells were cultured in TB medium with 1% glucose (v/v) and induced with IPTG and 0.5% glycerol (v/v). Based on antimicrobial activity results, the highest basal expression levels (un-induced) of mCherry-NisA was observed when LB or TBG was used as expression media (Fig. 7B, Off C). In contrast, the lowest basal mCherry-NisA expression levels or smallest inhibition zones were detected when expression was conducted in M9 and TB-G (Fig. 7B, Off C). After cleavage and purification, the correct mass (3352.514; m/z 671.71) for fully modified nisin was confirmed using ESI–MS (see Additional file 1: Fig. S11).

### Heterologous expression of class I epi-D like lanthipeptides

A clausin expression system was designed very similar to that of the NisA system described in the preceding sections. The ClausSTldhT7 expression strain (Fig. 1C) harbors theClausAprecursor peptide gene fused to the N-teminal His-tagged mCherry under the control of the STldh promoter as well as *clausB* placed under the control of the T7 promoter. A hNisP protease recognition site was engineered between the clausin leader- and clausin core peptide sequence for liberation of mature clausin or epidermin by NisP. mCherryClausA was co-expressed with ClausC (pClausCi) and ClausD (pClausDi) (Fig. 1C). As proof of principle for the utilization of the clausin modification machinery as a ‘plug and play’ system for the heterologous production of other epidermin-like lanthipeptides, the epiderminA precursor gene (*epiA*) was synthesized and fused to the gene fragment encoding the ClausA leader peptide (see Additional file 1: Fig. S9). yielding the EpiSTldhT7 expression strain (Fig. 1C).

**Fig. 8:**
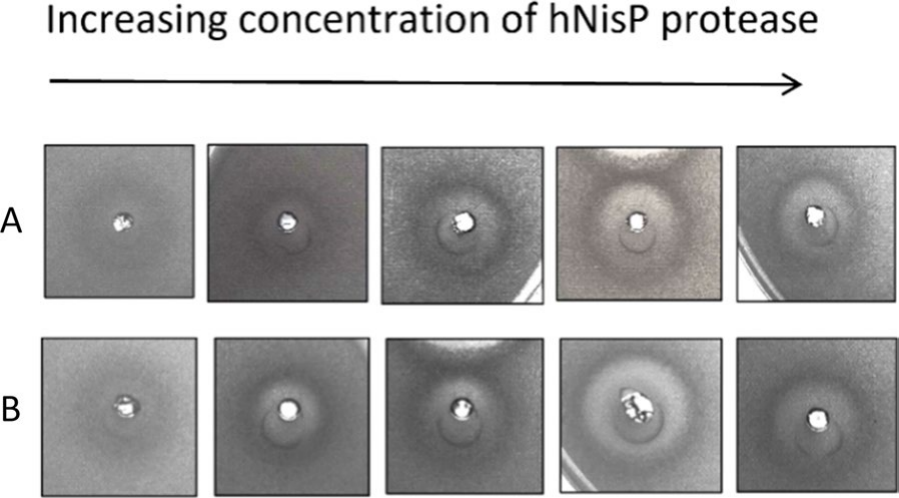
Antimicrobial activity and cleavage optimization of mCherry-PreLanthipeptides using hNisP. **A** mCherry-PreClausA purified from strain ClausSTldhT7 and **B** mCherry-PreEpiA purified from strain EpiSTldhT7 after cleavage for 20 h at 26 °C respectively. hNisP was added at concentrations 0, 1.1, 15, 120 and 300 ng/µL. After cleavage, 50 µL of mCherry fused Pre-Lanthipeptide was spotted from each cleavage reaction against *L. sakei*

**Fig. 9:**
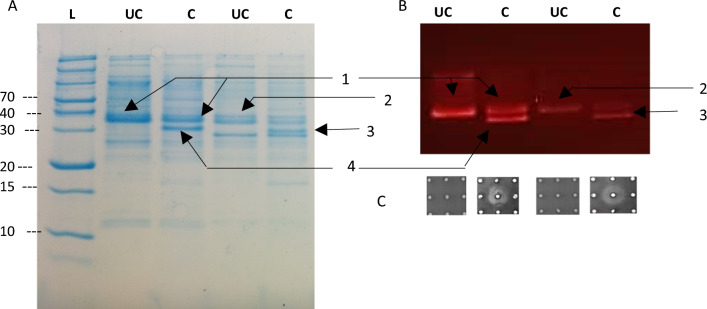
SDS-PAGE stained gels and mCherry fluorescent images representing cleavage of mCherry-PreClausA and mCherry-PreEpiA at optimal concentrations of hNisP. **A** Image representing mCherry-PreLanthipeptides in a Coomassie-stained gel and **B** image representing fluorescence of mCherry in gels before fixing and staining, with **C** antimicrobial activity shown against *L. sakei* of uncleaved and cleaved samples used for loading onto gels. *L* ladder, *UC* uncleaved, *C* cleaved; 1: Uncleaved mCherry-PreClausA; 2: Uncleaved mCherry-PreEpiA; 3: Cleaved mCherry-PreClausA; 4: Cleaved mCherry-PreEpiA

**Fig. 10:**
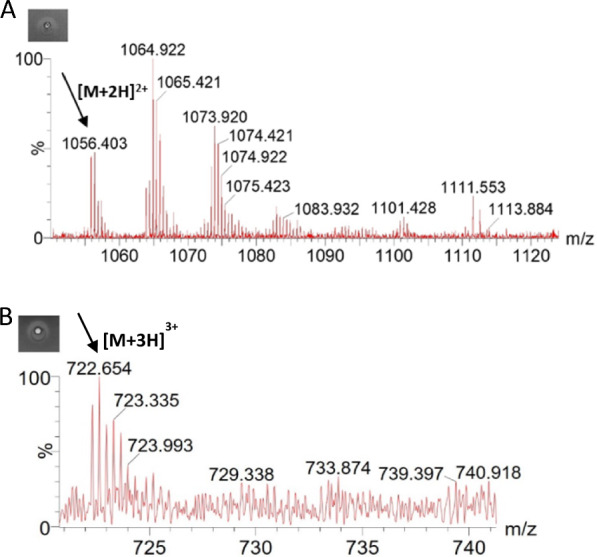
ESI–MS results for hNisP cleaved mCherry-PreLanthipeptides produced in this study. Mass spectrum of hNisP cleaved **A** mCherry-PreClausA and **B** mCherry-PreEpiA produced in *E. coli*. The fully dehydrated monoisotopic peaks indicated with an arrow in **A** and **B** correspond to mass measurements of 2110.79 Da (m/z 1056.4) for clausin carrying + 2 charges ([M/2 + 1.007276] predicted m/z 1054.94 [7× dehydrated]) and 2164.92 Da (m/z 722.65) for epidermin carrying + 3 charges ([M/3 + 1.007276] predicted m/z 722.34 [5× dehydrated]), respectively. Activity zones are representative of cleaved and extracted samples before ESI–MS analysis

Heterologously expressed mCherry-PreClausA and mCherry-PreEpiA were purified and cleaved with hNisP, resulting in the liberation of bioactive core peptides after 20 h of cleavage (Fig. 8). No antimicrobial activity was detected in the absence of hNisP cleavage (Figs. 7B and 8A). Antimicrobial activity for cleaved mCherry-PreClausA and mCherry-PreEpiA increased proportionally as hNisp cleavage concentrations increased from 1 to 120 ng/µL (Fig. 8). Cleavage activation (hNisP) of mCherry fused clausin and epidermin lanthipeptides could be visualized clearly using both fluorescent imaging and conventional Coomassie staining to track the migration of cleaved and uncleaved mCherry fused samples (Fig. 9). Additionally, background proteins could clearly be differentiated from uncleaved or cleaved mCherry fused peptides using fluorescent imaging (Fig. 9B). In the cleaved Pre-ClausA sample, two fluorescing bands were detected, indicating some incomplete cleavage at the protease concentration yielding optimal antimicrobial activity (Fig. 9B). Using the heterologous expression system optimized in this study, an approximate per liter yield reached 26 mg for mCherry fused Pre-ClausA and 23 mg for mCherry fused Pre-EpiA. Based on the size ratio of the mCherry-ClausA leader-to- clausin and epidermin core peptide segments (both ~ 14:1), final yields of approximately 1–2 mg of active clausin and epidermin would be generated when the mCherry+ClausA leader segments are removed. After cleavage and extraction, masses consistent with that of fully and partially modified clausin and epidermin was confirmed using ESI–MS (Fig. 10). Peaks with mass approaching the predicted masses of clausin (2107.86 Da; m/z 1054.94) and epidermin (2164.01 Da; m/z 722.34) were identified upon ESI–MS analysis of cleaved and extracted fusion peptides (Fig. 10A, B, Table 1). Variants with different dehydration states were detected, ranging from 7× (fully modified) to 5× dehydrations for clausin. The final mass of the peptides shown in Table 1, includes the loss of 46 Da as a result of the oxidative decarboxylation by ClausD, to yield a *S*-[(Z)-2-aminovinyl]-d-cysteine residue at the C-terminal ends of the respective peptides. For epidermin, a minor species of four dehydrations was found, but the dominant species was fivefold dehydrated (fully modified) (Fig. 10B and Table 1). This is the first report on the heterologous expression of modified and bioactive clausin and epidermin using the clausin modification machinery in *E. coli*.

**Table 1:** Predicted massess of epidermin-like lanthipeptides produced in this study, compared to those found in ESI–MS analyses

Peptide	Dehydrated	Predicted mass		Observed mass		Charge	Difference (Da)	Fig.
		(m/z)	(Da)	(m/z)	(Da)			
Clausin	7×	1054.937	2107.860	1056.403	2110.791	+ 2	2.931	Fig. 10A
	6×	1063.937	2125.860	1064.922	2127.829	+ 2	1.969	
	5×	1072.937	2143.860	1073.920	2145.825	+ 2	1.965	
Epidermin	5×	722.344	2164.010	722.654	2164.940	+ 3	0.940	Fig. 10B
	4×	728.344	2182.010	729.338	2184.992	+ 3	2.982	

The charges and the mass error shown as the absolute difference in Daltons are also indicated

Mass (Da) = m/z * 2 − 2 * 1.007276 when carrying 2+ charged ions or Mass (Da) = m/z * 3 − 3 * 1.007276 when carrying 3+ charges

## Discussion

Bacterial lanthipeptides with novel modes of action represent an inexhaustible source of new antibiotics as promising alternatives for existing traditional antimicrobial therapeutics due to their demonstrable high potency and ability to rapidly destroy target cells, including many drug-resistant targets [1, 18, 29, 43, 44]. To fully exploit the functionality of these novel lanthipeptides a systematic expression, purification and characterization process is required. In this study we constructed an efficient ‘plug and play’ heterologous expression system in *E. coli* for the production and purification of class I lanthipeptides. Screening a wide array of expression conditions has routinely been practiced for the optimal production of bacteriocins [20, 29, 33, 38, 45, 46]. Fusion of lanthipeptide structural genes to mCherry allowed us to investigate the impact of key factors that control the heterologous expression of class I lanthipeptides in *E. coli*. Using real-time in vivo fluorescence imaging combined with antimicrobial activity, we determined the impact of promoter arrangement and culture conditions (including choice of growth media and inducer agents) on the heterologous expression of nisin, clausin and epidermin.

The use of mCherry as a fusion partner provides several advantages beneficial for lanthipeptide production and bioprocess engineering [47, 48]. Of significance is enhanced lanthipeptide solubility in the cytosol, reduced potential toxic effects to the producing host and the ability to track the tuned expression of fused lanthipeptides in real-time and in vivo prior to purification and characterization steps. The ‘plug and play’ expression system designed here expresses mCherry-LanA (LanA, lanthipeptide precursor peptide) fusion gene products under control of the STldh promoter, providing the formation of bright purple colonies. The STldh promoter is a 135 bp promoter responsible for the conversion of sugar to lactate in the probiotic *E. mundtii* ST4SA, providing robust, high-level expression with cross-species compatibility and therefore, imparts several beneficial features for the development of an expression system [40–42].

Promoter selection for dual expression was also crucial for optimized mCherry-NisA production; in this case, the STldh-T7 promoter combination resulted in drastically improved mCherry-NisA fluorescence signals (strain NisSTldhT7) and improved antimicrobial activity of cleaved Pre-NisA in comparison to the ST1dh-STldh promoter combination (strain NisSTldhSTldh). Since both producing strains shared similar growth characteristics, we can only conclude that the STldh (MCS1) and T7 (MCS2) combination favored the expression of functional Pre-NisA. One possible explanation for the observed differences may be due to transcriptional interference (TI) due to the respective promoter arrangements in the mCherry-NisA constructs [49, 50]. Since the functional expression of lanthipeptides requires the co-expression of several genes from multiple promoters it is important to consider potential downstream interfering effects when choosing the respective promoters to drive the expression of protein coding-genes carried in heterologous expression plasmids. Based on results derived from fluorescence and activity data, we concluded that it was necessary to overexpress the mCherry and precursor peptide fusion constructs strongly, since mCherry is highly soluble and non-toxic to host cells [48]. Overexpression of the much larger lanthipeptide post-translational modification enzymes may require more extensive resources for its biosynthesis and was therefore, placed under control of the IPTG inducible T7 promoter for co-expression with mCherry-LanA (strain NisSTldhT7). We achieved approximately 24 mg of modified mCherry-fused NisA precursor per liter of *E. coli* culture, which corresponds to approximately 2–3 mg of final active product, and a twofold yield improvement compared to core lanthipeptide yields recorded with expression systems using GFP as fusion partner [37, 38]. Shi et al. [28] produced 24 mg/L of the NisA precursor peptide without fusion to a reporter gene or solubility enhancer. While we agree that the core peptide yields obtained are less it is important to stress that the main aim of our study was to construct a ‘plug and play’ system to produce and purify class I lanthipeptides in *E. coli*, using in vivo fluorescence as a platform to evaluate the expression of mCherry-fused lanthipeptides under various expression conditions. Additionally, our system provides several advantages in optimization of expression, including more effective PTMs because of increased peptide solubility when fused to mCherry and the ability to monitor proteolytic cleavage more accurately using the fluorescent properties of the mCherry-fusion. Our results provide a basis for further studies to optimize yields, and we are investigating the possibility to improve the expression and purification of NisA and novel derivatives using our system.

Using the NisSTldhT7 expression system the separate and combined effects of glucose, glycerol and IPTG addition to expression media, or their inclusion in expression media as inducer agents, on the heterologous expression of the mCherry-NisA fusion protein was monitored in vivo and in real-time. Glucose addition to pre-growth media resulted in significantly improved mCherry-NisA expression when cultures are grown to stationary phase. Naturally, the STldh promoter transcriptionally controls the *ldhA* (L-lactate dehydrogenase) gene responsible for the production of l-lactate as a fermentation by-product when metabolism transitions from exponential to stationary growth phase [41]. However, it was shown that the *ldhA* gene in *C. glutamicum* is positively regulated by the cAMP receptor protein-type global regulator GlxR [51]. Similarly, the widely available *lac* and *lacUV5* inducible promoters used in *E. coli* DE3 lysogens are controlled positively by the presence of high levels of cAMP and cAMP receptor protein (CAP) when glucose levels decrease in the expression media, also known as catabolite repression [30, 52]. Low background or un-induced expression levels can be maintained by supplementing the medium with glucose, also known as the glucose effect [52]. Glucose is easily metabolized by cells and thus an ideal substrate for cell growth in *E. coli* during the induction phase. While glucose can be used effectively as a carbon source, studies have shown that the presence of glucose in cells represses transcription from the widely used *lac* promoter in IPTG or lactose inducible expression systems via catabolite repression [30, 52–54]. The use of glucose-controlled expression may be especially useful in preventing high basal expression levels of the much larger lanthipeptide PTM enzymes compared to precursor peptides. Glycerol as a carbon source, either as a supplement to pre-growth media (TBG) or as inducer agent at the induction growth phase (TB-G), significantly improved the expression of mCherry-NisA. Glycerol is a heavier carbon source compared to glucose and its metabolism causes cAMP levels to rise, thereby stimulating increased transcription levels from the STldh or lac promoters in *E. coli* BL21 [55–57]. As such, when cultures inch closer to stationary phase, any available glucose in the growth medium is consumed first before the utilization of glycerol as an alternative carbon source which can achieve enhanced biomass/protein yields [58, 59]. These results provide a strong indication that glycerol may be used as a soft or tunable inducing agent to reduce metabolic stress levels of the host. For example, the use of harsh induction agents such as IPTG to fully induce the promoter may lead to a metabolic or product burden on the inducer cells and therefore, the expression of the recombinant proteins may be limited due to toxicity or incorrect processing.

The effects of growth medium composition at or during the induction phase are critically important when optimizing the heterologous expression of bacteriocins in *E. coli* BL21 [20]. Pre-mature induction of the promoter or combination of promoters used for expression may lead to lower cell growth rates and product yields or may interfere with the normal functioning of the host cells due to metabolic burden [59]. Studies examining the expression of bacteriocins in *E. coli* under the control of inducible promoters have utilized both complex (such as LB or BHI medium) and minimal (such as M9 medium) growth media [60–63]. Unsurprisingly, LB is the most used medium as it is easy to prepare and generally provides an abundance of nutrients and trace elements to support growth. However, *E. coli* cell densities obtained with LB are low, which in turn affects bacteriocin yield and its undefined nature can be problematic. Similarly, the decreased antimicrobial activity observed when M9 minimal media is used as an expression medium could be attributed to poor biomass accumulation. Commercially available complex growth mediums for culturing *E. coli* such as LB, BHI, or nutrient broth may contain low or high levels of inducer agents (for example, lactose) or the presence of transcription enhancers such as cAMP that may lead to enhanced basal level expression of foreign proteins [30]. This may explain the observed significant increase in mCherry-NisA fluorescence when un-induced cells were grown in LB and BHI, despite the addition of glucose to pre-growth media. In addition, increased un-induced basal expression of bioactive mCherry-NisA in LB or BHI was achieved after purification and hNisP cleavage. The choice of media can also impact the functionality of modifying enzymes during heterologous expression in *E. coli*. A recent study used commercially sourced LB or TB medium to demonstrate differences in modifying enzyme activity of five lanthipeptide post-translational peptide modification enzymes in *E. coli* [64]. However, the study only focussed on the modifying activity of single enzymes as opposed to the co-expression of multiple peptide modification enzymes required to produce class I lantibiotics.

Overall, the best-producing conditions for mCherry-NisA using the plasmid system constructed in this study was when cells were cultured in TB-G (TB with glycerol and IPTG as inducers) or TBG (TB supplemented with glycerol, and IPTG as inducer) compared to M9, LB and BHI. In addition to enhancing cell biomass, it has been reported that supplementing growth media with increasing amounts of yeast extract increases un-induced background expression from the T7 lac promoter due the presence of cAMP or other compounds that can enhance transcription [65, 66]. While the preparation of TB-G and TBG includes the addition of more than 4× the amount of yeast extract compared to commercially sourced LB broth, they were the best-producing conditions for mCherry-NisA. It is, however, important to consider optimizing conditions for different peptides, since Pal and Srivastava [61] reported that the best-producing conditions for plantaricin E in *E. coli* were in LB, compared to TB. In our system supplementing the growth medium with glucose to reduce background mCherry expression and switching to a poor carbon source (glycerol) in the final growth cycles yielded the highest level of induction. Future studies need to include an investigation into the percentage of unmodified, partially modified and modified class I lanthipeptides produced in different growth media and inducing conditions. The focus of this study was first to determine the optimal growth and expression conditions to produce mCherry-fused nisin in *E. coli*, using in vivo fluorescence imaging and antimicrobial activity as tools, and then set the platform for investigations into the extent of modification and its suitability for other lanthipeptides.

Having identified and optimized key factors that are important for the stable and efficient expression of NisA in our heterologous expression system, we attempted the expression of two lanthipeptides that belong to a different subclass. The expression system employs a minimal gene cluster by co-expressing four of eleven genes [33] required for full Pre-ClausA biosynthesis in a three-plasmid-based system. We hypothesized that the biosynthetic machinery of clausin may be used in a prototypical heterologous expression system to produce lanthipeptides within the same subclass. A NisP protease recognition site was engineered between the clausin leader- and clausin or epidermin core-peptides for release of mature core peptides. Using the epidermin-like lanthipeptide heterologous expression system described here, mCherry-PreClausA- and -PreEpiA fusions were successfully expressed, cleaved and purified yielding antimicrobial activity against *L. sakei*. We show that heterologously produced NisP can cleave the leader peptide of ClausA, thus further expanding the substrate specificity of NisP. Proteolytic cleavage with hNisP resulted in the traceless removal of the core peptides from the mCherry-leader peptide fusions. In future studies, we intend to analyze whether EpiP, the wild-type serine protease responsible for cleavage activation of *epiA*, can process heterologously produced Pre-EpiA using the expression system constructed in this study [37, 67].

This is the first report of clausin heterologous expression using the clausin modification machinery in *E. coli*. In addition, we have demonstrated for the first time the functional expression of epidermin fused to the ClausA leader peptide. It must be pointed out that we have demonstrated the promiscuity of the clausin biosynthetic enzymes in producing epidermin by fusion of the ClausA leader to the *epiA* core peptide gene. The C-terminus of Pre-ClausA and Pre-EpiA undergoes oxidative decarboxylation to form *S*-[(Z)-2-aminovinyl]-d-cysteine (AviCys) and is catalyzed by the oxidoreductase LanD (ClausD in this study). Several peptides, including lantibiotics, containing AviCys residues, have been reviewed as potential drug candidates due to their highly potent biological activities, ranging from antimicrobial activity against methicillin-resistant *S. aureus* (MRSA) to anticancer activity against mouse leukemia cells [10, 68]. To our knowledge, clausin and epidermin have been exclusively produced by using wild-type hosts through the purification of mature peptides from the producer supernatant [1, 69–73]. However, the epidermin-like heterologous expression system constructed in this study, not only offers rapid production of mature lanthipeptides in *E. coli* (expression for 1 day versus 10–14 days in native producers) but also could serve as a platform for future studies into the characterization and function of class I epidermin-like lanthipeptides. In subsequent strain optimization experiments the expression systems developed herein will be used to investigate whether the inclusion of optimized tRNA^Glu^ sequences and glutamyl-tRNA synthetase can increase efficiency of ClausB in *E. coli* [34, 35].

## Conclusion

We succeeded in developing a plug and play expression system to produce various class 1 lanthipeptides that relies on in vivo fluorescence monitoring of mCherry-fused lanthipeptides to evaluate expression. We developed two novel mCherry-fusion-based heterologous expression systems to produce two different subgroups of class I lanthipeptides. Fully modified Pre-NisA, Pre-ClausA and Pre-EpiA fused to the mCherry fluorescence gene were purified from the Gram-negative host *E. coli* BL21 (DE3). Through fusion, the robust fluorometric properties of mCherry allowed a substantial reduction in optimization time, since expression could be monitored in real-time, without the need for extensive and laborious purification steps or the use of in vitro activity assays. The mCherry-NisA expression system was used to elucidate the effect of culture media composition, promoter arrangement, and culture conditions including choice of growth media and inducer agents on the heterologous expression of the class I lanthipeptides. Our results show that the expression conditions determined for nisin could be directly transferred to produce active clausin and epidermin in *E. coli*. We determined that the addition of 1% glucose to pre-growth media can effectively control the un-induced background basal expression levels, that may in turn negatively affect plasmid stability or place a heavy metabolic burden on the host cell resources prior to the production phase. The highest mCherry-Lan production levels were achieved when using glycerol and IPTG as inducer agents and TB as culture medium (without prior addition of glycerol). The heterologous expression systems constructed in this study may be used in subsequent investigations for the scalable expression of novel NisA derivatives, or novel genome mined derivatives of clausin. The full potential of the heterologous expression systems used in this study will have to be evaluated by the screening of smaller fluorescent proteins. This may require the inclusion of secretion signals to export the fused proteins. This may improve production yields and the purification of lanthipeptides.

## Materials and methods

### Bacterial strains and growth conditions

Bacterial strains and plasmids used in this study are listed in Additional file 1: Table S1. All subclonings were performed in *E. coli* DH5α [74] and protein expression in *E. coli* BL21 (DE3). *Escherichia coli* strains were grown at 37 °C in Luria–Bertani (LB) broth (Biolab Diagnostics, Midrand, South Africa) agitated at 200 rpm, or on LB agar (1.5%, w/v) plates. *Escherichia coli* cultures containing plasmids (Additional file 1: Table S1) were supplemented with either 50 µg/mL kanamycin (Kan), 25 µg/mL chloramphenicol (Cm) or 100 µg/mL ampicillin (Amp). The wild-type NisinA producer, *Lc. Lactis* QU2, was grown statically at 30 °C in M17 broth (Biolab Diagnostics) or on solid media supplemented with 0.5% (w/v) glucose. *Bacillus clausii* AD1 [41] was grown in Brain Heart Infusion (BHI) and incubated at 37 °C. The probiotic strain *Enterococcus mundtii* ST4SA was cultured statically at 30 °C in MRS broth (Biolab Diagnostics). The indicator strain, *Lactobacillus sakei* DSM 20017, was grown without shaking at 30 °C in MRS broth or on MRS agar plates.

### DNA manipulation techniques and cloning procedures

Recombinant DNA manipulations and general cloning procedures were performed as described by Sambrook and Russell [75]. DNA restriction and modification enzymes were from New England Biolabs (NEB, Ipswich, MA, USA) and were used according to the suppliers’ protocols. Oligonucleotides (Additional file 1: Table S2) for insert amplification were from Inqaba Biotechnical Industries (Pretoria, South Africa) and were designed using Snapgene® software (Insightful Science; available at snapgene.com). PCR reactions were performed using Q5 high-fidelity DNA polymerase (NEB, Ipswich, MA, USA) according to the manufacturer’s instructions and a GeneAmp PCR system 9700 (ABI, Foster City, CA, USA). DNA fragments were purified from agarose gels using the Zymoclean™ gel DNA recovery kit (Zymo Research Corporation, Irvine, CA, USA) or the GeneJet PCR purification kit (Thermo Fisher Scientific Baltics UAB, Vilnius, Lithuania). Plasmid DNA was purified from *E. coli* transformants using the PureYield™ plasmid miniprep system (Zymo Research Corporation) and sequenced at the Central Analytical Facility (CAF) of Stellenbosch University. DNA concentrations were determined using the BioDrop µLite+ (Biochrom, Cambridge, UK). Genomic DNA (gDNA) of *Lc. Lactis* QU2, *B. clausii* AD1 and *E. mundtii* ST4SA were isolated using the ZR Fungal/Bacterial DNA miniprep kit (Zymo Research Corporation) following the manufacturer’s instructions. Electrocompetent *E. coli* cells were prepared as described previously [74] and transformed using the Bio-Rad Gene Pulser electroporation system (Bio-Rad Laboratories, Hercules, CA, USA).

### Construction of heterologous expression vectors and lanthipeptide expressing strains

The pRSTc*nB nisin-like lanthipeptide expression backbone plasmid was constructed as follows. First, plasmid pNZSTldhMCS1-2 was digested with HpaI and XhoI, generating a 1114 bp fragment containing the *E. mundtii* ST4SA lactate dehydrogenase (STldh) promoter with a hexahistidine tag in the first multiple cloning site (MCS1), and the IPTG inducible T7 promoter in MCS2. The 1114 bp fragment and pRSFDuet-1, also digested with HpaI and XhoI were purified from an agarose gel (0.8%, w/v) and ligated using T4 DNA ligase according to the manufacturer’s instructions. The resulting plasmid (pRSTldhMCS1-2) was then double digested with BamHI/NotI and ligated to a 752 bp *mGFP5* gene fragment (digested from pRSFGFP). The construct (pRSTldhGFP) contained a C-terminal WELQ site. To incorporate the *mCherry* fluorescence gene, a 698 bp BamHI/PstI fragment was digested from plasmid pNZSTldhCherry and cloned into pRSTldhGFP digested with the same restriction enzymes. The construct (pRSTldhCherry) contained an N-terminal His-tag fused to *mCherry* with a C-terminal WELQ site under control of the STldh promoter. Finally, the 2982 bp *nisB* dehydratase gene was amplified from *Lc. Lactis* QU2 gDNA using primer pair nisB1/nisB2 that contained the BglII/XhoI digestion sites and ligated with the double-digested pRSTldhCherry plasmid to yield the nisin-like backbone plasmid pRSTc*nB (see Additional file 1: Fig S4). The ribosomal binding site (RBS) of the STldh promoter (5′-GAAAGG-3′) is located 8 bp from the translational start site of the *mCherry* gene and a transcription terminator is located after MCS2 [40]. Detailed plasmid maps of the expression vectors used in this study are described in Additional file 1: Figs. S4–S9. The integrity of all plasmid constructs was verified by restriction digests, PCR and DNA sequencing using respective primer combinations (Additional file 1: Table S2). *E. coli* DH5α electrocompetent cells were used for all initial cloning reactions.

The pRSSc*nB backbone plasmid was constructed to incorporate a second STldh promoter in MCS2 of pRSTc*nB (Additional file 1: Fig. S5, Table S1). The STldh (156 bp) promoter was amplified from *E. mundtii* ST4SA gDNA using primer pair STldh1/STldh2, incorporating NotI and BglII digestion sites. The double-digested NotI/BglII STldh promoter fragment was then cloned into pRSTc*nB digested with the same two restriction enzymes. The new construct, pRSSc*nB, lacked the T7 promoter in MCS2 (see Additional file 1: Fig. S5).

To construct the mCherry-NisA fusion vectors, the *nisA* gene was PCR amplified using *Lc. Lactis* QU2 template gDNA and primers nisA1/nisA2 (Additional file 1: Table S2). The *nisA* gene fragment was cloned into pRSFDuet-1 as a BamHI/HindIII fragment. The 174 bp *nisA* gene (original ASPR, *nisP* (hNisP) cleavage site) was then digested out with PstI/NotI and ligated into the corresponding sites in pRSTc*nB (MCS1-STldh promoter; MCS2-T7) and pRSSc*nB (MCS1 and MCS2-STldh), yielding pRSTcnA*nB (see Additional file 1: Fig. S6A) and pRSScnA*nB, respectively (see Additional file 1: Fig. S6B). Both resulting expression vectors carried the N-terminally His-tag fused mCherry with a C-terminal WELQ site under control of the STldh promoter in MCS1 (see Additional file 1: Fig. S6C, D). The C-terminal of mCherry (with WELQ site) was directly fused to the N-terminal region of nisin. The pRSSc*nB (see Additional file 1: Fig. S5A) backbone plasmid was constructed to incorporate a second STldh promoter in MCS2, thereby replacing the T7 IPTG inducible promoter in MCS2 of pRSTc*nB. This placed both the mCherry-pLanthipeptide fusion fragment and the *nisB* gene under control of STldh, while *nisC* cyclase expression is controlled by the T7 promoter in the low copy number vector pACYC-Duet1 for both mCherry-nisA vector combinations.

To construct the mCherry-NisA lanthipeptide expression strains, the respective pRSTcnA*nB (see Additional file 1: Fig. S6A) and pRSScnA*nB (see Additional file 1: Fig. S6B) constructs and pACYC-NisCi were co-transformed into electrocompetent *E. coli* BL21 (DE3) cells and plated onto LB agar, supplemented with Kan and Cm, and incubated overnight at 37 °C. Bright pink single colonies were isolated by re-streaking onto fresh LB plates and used in subsequent expression experiments. Strain names and constructs contained within each strain are illustrated in Fig. 1. For the NisSTldhSTldh (Fig. 1B) mCherry-NisA expression system, the mCherry-NisA fusion fragment and the *nisB* (dehydratase) gene were placed under control of the STldh promoter. The T7 promoter in the low copy number vector pACYC-Duet1 controls *nisC* (cyclase) expression in both the NisSTldhT7 and NisSTldhSTldh expression systems (Fig. 1).

To construct the backbone of the epidermin-like lanthipeptide heterologous expression plasmid, the *clausB* dehydratase gene was amplified from the genome of *B. clausii* AD1 by using the PCR primer pair clausB1/clausB2. The 3156 bp *clausB* amplicon was cloned into MCS2 of pRSTldhCherry, using BglII and XhoI, to yield the pRSTc*cB (see Additional file 1: Fig. S7) backbone plasmid. Similarly, the *clausC* (cyclase) and *clausD* (decarboxylase) modification genes were amplified by PCR using primer pairs clausC1/clausC2 and clausD1/clausD2, respectively, and *B. clausii* AD1 gDNA as template. Amplicons of *clausC* (1299 bp) were digested with EcoRV/XhoI and cloned into MCS2 of pACYCDuet-1 to produce pClausCi (see Additional file 1: Fig. S8B–E). Fragments amplified from *clausD* (510 bp) were digested with BamHI/HindIII and cloned into the MCS of pKSBluescript to produce pClausDi (see Additional file 1: Fig. S8C–F).

Construction of the clausin and epidermin core-peptides fused to the original clausin leader peptide and the ASPR NisP (hNisP) cleavage site was achieved using overlay fusion PCR. The *epiA* gene was chemically synthesized and PCR amplified using primer pair epiAFor/epiARev. The forward primers used to amplify the Pre-ClausA (ClausNisPLanOL) and Pre-EpiA (EpiANisPOL) core peptide segments incorporated a 5′ overhang homologous to the ASPR (12 bp) hNisP cleavage site and the 3′region of the clausin leader. The 54 bp clausin leader was amplified using *B. clausii* AD1 gDNA as template with primers ClausLFor/ClausLNisPRevOL, with the reverse primer incorporating the 12 bp nucleotide sequence coding for the hNisP ASPR protease site. For overlay fusion PCR the clausin leader PCR product and the respective clausin/epidermin core peptide PCR products were combined and amplified using primers ClausLFor/ClausNotIRev for *clausA* or ClausLFor/EpiANotIRev. The final fused ClausA and EpiA precursor lanthipeptides were digested with PstI/NotI and ligated into the corresponding sites in pRSTc*cB to give pRSTccA*cB (see Additional file 1: Fig. S8A–D) and pRSTceA*cB, respectively (see Additional file 1: Fig. S9). In both resulting vectors, the respective mCherry-ClausA (see Additional file 1: Fig. S8D) and mCherry-EpiA (see Additional file 1: Fig. S9B) gene fragments were cloned downstream of the STldh promoter, while *clausB*, *clausC* and *clausD* were placed under the control of T7 IPTG inducible promoter (Fig. 1).

To construct the epidermin-like lanthipeptide heterologous expression strains, the respective lanthipeptide containing plasmids pRSTccA*cB (see Additional file 1: Fig. S8A) and pRSTceA*cB (see Additional file 1: Fig. S9A) were each co-transformed with plasmids pClausCi (see Additional file 1: Fig. S8B–E] and pClausDi (see Additional file 1: Fig. S8C–F) into electrocompetent *E. coli* BL21. Transformed cells were plated onto LB agar supplemented with Kan, Cm and Amp and incubated as described previously for isolation of bright red single colonies to be used for expression experiments. In both the ClausSTldhT7 and EpiSTldhT7 expression systems (Fig. 1C), the respective mCherry-ClausA (see Additional file 1: Fig. S8D) and mCherry-EpiA (see Additional file 1: Fig. S9B) gene fragments were cloned downstream of the STldh promoter, while *clausB*, *clausC* and *clausD* were placed under the control of T7 IPTG inducible promoter (Fig. 1). The ASPR cleavage site of hNisP present in Pre-NisA constructs was introduced into the leader peptide of ClausA and EpiA via fusion PCR for in vitro cleavage activation of heterogously produced lanthipeptides.

### Determination of mCherry expression

In vivo fluorescence of *E. coli* BL21 cultures was recorded using the Tecan Spark™ 10 M multimode microplate reader connected to Tecan i-control 1.6 software (Tecan Group Ltd, Mӓnnedorf, Switzerland). *Escherichia coli* BL21 (DE3) cells transformed with pRSF Duet-1 lacking *mCherry* (thus non-fluorescent) and pACYC Duet-1, respectively, served as a negative control strain. *Escherichia coli* BL21 cells that expressed mCherry-NisA were co-transformed with plasmids pRSTcnA*nB and pACYC-NisCi (Fig. 1A). Cultures were grown in terrific broth without glycerol (TB-G) or in terrific broth containing 0.5% (w/v) glycerol (TBG), and was supplemented with Kan and Cm, for 12 h. One millilitre of the culture was inoculated into 50 mL freshly prepared TB-G or TBG, kept at 37 °C. The 50 mL cultures were supplemented with glucose (1.0%, w/v) or without glucose. All cultures were incubated at 37 °C under constant aeration until an OD_600nm_ of 0.6. Thio-B-d-galactopyranoside (IPTG; 0.15 mM) or glycerol was added to the cultures, and they were incubated at 26 °C for 24 h. Glycerol was not used as inducer agent when TBG was used as an expression medium. The expression of mCherry-NisA of each 50 mL culture was fluorometrically measured in relative fluorescence units (RFUs) at 587 nm (excitation) and 610 nm (emission). Measurements were taken at 26 °C at the time of induction (4 h) and at 6 h, 8 h, and 24 h. A volume of 200 µL of each culture was pipetted into a black 96-well clear bottom microplate (Perkin Elmer, USA) and imaged using a gain of 54. Immediately prior to fluorescence measurements, samples were shaken for 5 s. All experiments were done with three biological repeats and measurements taken in triplicate.

### Optimization of mCherry-fused pNisin expression in different growth media

Culturing and expression media were supplemented with glucose (1.0%, w/v) throughout the experiment. The expression of mCherry-NisA was optimized in LB, BHI, TBG, TB-G and M9 culture media. M9 minimal media was prepared as described by Sambrook and Russel [75]. Expression efficiency (fluorescence emission) was determined using the Tecan Spark™ 10 M multimode microplate reader. Basal (un-induced) and induced antimicrobial activity levels of heterologously produced NisA in each culture medium were assessed against *Lb.* sakei grown in MRS medium by agar well diffusion assay. *E. coli* BL21 cells were co-transformed with mCherry-NisA production plasmids pRSTcnA*nB and pACYC-NisCi. Single colonies were used to inoculate 10 mL of each culture media, supplemented with Kan and Cm, and incubated for 12 h. One milliliter of the 12 h old cultures was used to inoculate 50 mL of the respective freshly prepared pre-warmed media and incubated at 37 °C on an orbital shaker (225 rpm). At an OD_600nm_ of 0.6 all cultures were induced with 0.15 mM IPTG and 0.5% glycerol (v/v) followed by incubation at 26 °C for 24 h. The in vivo expression of mCherry-NisA of each 50 mL culture was fluorometrically measured as described elsewhere. All experiments and measurements were performed in triplicate.

### Peptide purification and extraction

Induced cells were harvested (7025×*g*, 20 min at 4 °C) and cell pellets subjected to at least five 20-min freeze–thaw cycles at − 80 °C to increase lysis efficiency. Cell pellets were then resuspended in 10 mL start buffer (SB, 50 mM Tris, 500 mM NaCl, 10% glycerol, pH 8.0), supplemented with lysozyme, RNaseI and DNaseI to final concentrations of 1.0 mg/mL, 10.0 µg/mL and 5.0 µg/mL, respectively, followed by incubation on ice for 30 min and sonication. Cell suspensions were sonicated five times at 50% power output, 2 s pulse, and 10 s pause for 30 s using the Omni Ruptor 400 (Ultrasonic Homogenizer, Omni International). Cell debris was separated from the soluble lysate by centrifugation for 60 min at 15,870×*g* at 4 °C. The His-tagged mCherry-lanthipeptide fusion proteins were applied to 1.0 mL HisTrap HP columns (GE Healthcare, PA, USA) and purified by IMAC. Ni^2+^-NTA superflow resin was used (ThermoFisher Scientific, IL, USA) according to the suppliers’ instructions. The cell-free lysates as well as the Ni^2+^-NTA superflow resin were equilibrated to 10 mM imidazole in SB buffer (SB10, start buffer containing 10 mM imidazole) prior to loading the column for IMAC purification using the Äkta purifier system (Amersham Biosciences). Loaded columns were washed with SB10 and eluted off the column using SB250 (SB buffer containing 250 mM imidazole). Eluted His-tagged fusion proteins were desalted for optimal hNisP cleavage using anion exchange chromatography (DEAE Sepharose fastflow resin, Cytiva, Marlborough, USA). His-tagged mCherry fused lanthipeptides were diluted 30× in 50 mM Tris (pH 8.3) before loading onto a 1 mL column. An increase in salt concentration was used to wash the column and to elute proteins at 10 mM and 150 mM NaCl, respectively. The concentrations of mCherry-fused lanthipeptides were determined using a BCA protein assay (ThermoFisher Scientific, IL, USA) and the BioDrop µLite (Cambridge, UK).

To remove mCherry from heterologously produced lanthipeptides, samples digested with hNisP (120 ng/µL for 24 h) were subjected to a second His-tag purification step. hNisP digested samples were adjusted to a final imidazole concentration of 10 mM and loaded onto Ni^2+^-NTA columns pre-equilibrated with SB10. The lanthipeptide containing eluent was collected and columns washed with PBS, SB10 and 30% isopropanol to elute non-specifically bound lanthipeptides. The eluent was dried using a SpeedyVac vacuum concentrator and stored at − 20 °C for subsequent use. Alternatively, hNisP cleaved samples were extracted using 1-butanol by adding the solvent to the sample in a 1:1 ratio. To separate the extracts, the mixture was kept stirring at room temperature for 2 h followed by centrifugation (3000×*g*, 5 min) at room temperature. Recovered lanthipeptide extracts contained in the upper organic layer were obtained and dried using a SpeedyVac vacuum concentrator and stored at − 20 °C for further applications.

### Antimicrobial activity tests

The antimicrobial activity of nisin, clausin and epidermin was assessed against *Lb. sakei* grown in MRS media by agar well diffusion assay. Twenty microlitres of fresh overnight *Lb. sakei* culture was inoculated into 100 mL pre-cooled MRS agar (1.0%). This mixture was added to a 145 × 20 mm Petri dish with a sterilized 96-well plate placed into the molten agar to create wells. The plates were allowed to cool and kept in a biosafety cabinet for 15 min to allow excess moisture to evaporate. All mCherry-fused lanthipeptide samples were cleaved with hNisP and set to a 50 µL final volume before loading into wells and incubation at 30 °C. For all activity tests, a positive control of 100 µL of 100 µg/mL (40 mg/mL stock solution in 0.05% acetic acid) Nisaplin (Sigma Life Science, Darmstadt, Germany) and a negative control of undigested mCherry-fused lanthipeptides were spotted. Antimicrobial activity was observed as a clear zone of growth inhibition and all tests were performed in triplicate with samples taken from at least three different expression batches.

### Optimization of protease digestion

mCherry-fused lanthipeptides were cleaved with heterologously produced nisin protease (hNisP) at 37 °C for 4 h and 20 h. hNisP expressed in *E. coli* BL21 (DE3) was added at final concentrations of 0.01 ng/µL, 1.16 ng/µL, 15 ng/µL, 120 ng/µL and 300 ng/µL in 50 µL reactions. Protein concentrations of mCherry-fused lanthipeptides were standardized to 1 mg/mL final concentrations, except for cleavage of mCherry-NisA fractions purified from different culture media, which was adjusted to 0.1 mg/mL (induced) and 0.05 mg/mL (un-induced). Protease inhibitors were used to stop reactions. Plates were incubated overnight at 30 °C and antimicrobial activity tested against *Lb. sakei* by agar well diffusion assay. Cleaved mCherry-fused lanthipeptides were visualised by fluorescence imaging and SDS-PAGE (tricine sodium dodecyl sulfate polyacrylamide gel electrophoresis. SDS-PAGE was used to visualize mCherry fluorescence after cleavage and separation from fused lanthipeptides. All mCherry-containing samples were processed with 15% gels according to standard protocols [75]. Samples were incubated at 37 °C for 30 min in to visualize mCherry fluorescence after electrophoretic separations. An aliquot of 7 µL of each sample was loaded into wells and gels were run in an ice bucket to aid separation of lanthipeptides. A light box coupled with a UV light and the In vivo Imaging System (IVIS, Caliper, USA) was used for visualization of mCherry fluorescence. After fluorescence imaging, all gels were washed with dH_2_O before fixation in 5% (v/v) glutaraldehyde for 1 h. After fixing, gels were washed in dH_2_O once more and visualized by staining with blue silver Coomassie stain as reported elsewhere.

### LC–ESI–MS sample preparation

For liquid chromatography-electrospray ionization-mass spectrometry (LC–ESI–MS) analysis of cleaved/extracted lanthipeptides, lyophilized extracts were reconstituted in acetonitrile (ACN/H_2_O/triflouro-acetic acid (50:50:0.1%). Active samples were concentrated by freeze drying or by using a SpeedyVac vacuum concentrator before ESI–MS analysis (CAF, Stellenbosch, South Africa). A lanthipeptide containing a sample solution volume of 2–5 µL was injected into a Waters Synapt G2 mass spectrometer (Waters Corporation, Miliford, USA) via a Waters Acquity UPLC using direct infusion. 0.1% formic acid in 50% acetonitrile/water (v/v) was used as ESI solvent at a flow rate of 300 µL/min. The analytes were subjected to a cone voltage (CV) of 25 V, capillary voltage of 2.5 kV, desolvation gas (N2) of 650 L/h, and source and desolvation temperatures were set to 120 °C and 275 °C, respectively. Positive mode scanning through m/z = 100–2000 in continuum mode (0.2 scans p/s) was used for data collection. All MS data generated were analysed using MassLynx v4.1 SCN 714 and the MaxEnt 3 algorithm.

### Supplementary Information

**Additional file 1: Figure S1.** Growth curves of *E. coli* BL21 strains NisSTldhSTldh and NisSTldhT7 expressing mCherry-NisA. Each vector with the respective promoter combinations was co-transformed with pACYCNisCi. The negative non-fluorescent control expression strain (Control) was transformed with empty pRSF-Duet 1 and pACYCNisCi. STldhSTldh = STldh promoter used for expression of mCherry-NisA and NisB; STldhT7 = STldh used for expression of mCherry-NisA and T7 promoter for NisB. **Figure S2.** Growth profiles of strain NisSTldhT7 expressing mCherry-NisA under various culturing and induction conditions. Cultures were grown in terrific broth supplemented without (**A**) and with (**B**) glucose (1% v/v) for 24 h. Control cultures were transformed with empty pRSF Duet-1 (without mCherry) and pACYC Duet-1 plasmids, while recombinant *E. coli* BL21 were transformed with pRSTldhCherryPrenisinNisB and pACYC-NisC plasmids. Culturing and induction conditions included: (−IPTG; −GlyM; −GlyIn) no induction; (−IPTG; +GlyM; −GlyIn) no IPTG induction with glycerol (0.5% v/v) added to pre-growth media; (−IPTG; −GlyM; +GlyIn) no IPTG induction but induced with glycerol (0.5% v/v); (+IPTG; +GlyM; −GlyIn) induced with 0.15 mM IPTG and glycerol added to pre-growth media; (+IPTG; −GlyM; +GlyIn) induced with 0.15 mM IPTG and glycerol; (+IPTG; −GlyM; −GlyIn) induced with 0.15 mM IPTG. IPTG = Thio-B-d-galactopyranoside; GlyM = glycerol presence in pre-growth media; GlyIn = glycerol used as inducer agent; +: added and −: not added. **Figure S3.** Growth profiles of strain NisSTldhT7 expressing mCherry-NisA in M9 minimal media, LB, BHI, TBG (TB containing 0.5% glycerol), TB-G (TB without 0.5% glycerol), **A** induced with 0.15 mM IPTG and 0.5% glycerol (v/v) and **B** un-induced. **Figure S4.** **A** pRSTc*nB backbone plasmid map. **B** Close up view of gene fragments. Relevant plasmid elements, promoters, genes and restriction sites are shown. Lanthipeptide genes are inserted at PstI/NotI restriction enzyme sites. **Figure S5.** **A** pRSSc*nB backbone plasmid map. **B** Close up view of gene fragments. Relevant plasmid elements, promoters, genes and restriction sites are shown. Lanthipeptide genes are inserted at PstI/NotI restriction enzyme sites. The T7 promoter in MCS2 was replaced with a second STldh promoter using NotI/BglII restriction enzyme sites. **Figure S6.** **A** pRSTcnA*nB and **B** pRSScnA*nB mCherry-nisA heterologous expression plasmids. **C**, **D** Linearized views of gene fragments shown in A and B, respectively. Relevant plasmid elements, promoters, genes and restriction sites are shown. **Figure S7.** **A** pRSTc*cB backbone plasmid map. **B** Close up view of gene fragments. Relevant plasmid elements, promoters, genes and restriction sites are shown. Lanthipeptide genes are inserted at PstI/NotI restriction enzyme sites. **Figure S8.** **A** pRSTccA*cB mCherry-PreClausA heterologous expression plasmid, **B** pClausCi clausC cyclase expression plasmid and **C** pClausDi clausD expression plasmid. **D**, **E** and **F** Close-up and linearized views of gene fragments shown in A, B and C, respectively. Relevant plasmid elements, promoters, genes and restriction sites are shown. **Figure S9.** **A** pRSTceA*cB backbone plasmid map. **B** Close-up view of gene fragments. Relevant plasmid elements, promoters, genes and restriction sites are shown. Lanthipeptide genes are inserted at PstI/NotI restriction enzyme sites. **Figure S10.** Differentiation of mCherry-NisA production based on color produced by host cells in spent expression media carrying the promoter arrangement described for strains (**1**) NisSTldhSTldh and (**2**) NisSTldhT7, respectively, after 20 h incubation. **Figure S11.** Mass spectrum of hNisP cleaved mCherry-NisA produced in *E. coli*. Activity zones are representative of cleaved and extracted samples before ESI–MS analysis. **Figure S12.** Visual representation of color differentiation of cell pellets produced when cultures are kept uninduced (off) and when induced (on). **Table S1.** Bacterial strains and plasmids used in this study. **Table S2.** Oligonucleotides utilized in this study.

## Data Availability

All dataset(s) generated and/or analysed during this study are included in the published article and the additional file.

## Abbreviations

AA: Amino acid
LanB: Dehydratase
LanC: Cyclase
LanM: Bifunctional synthetase
Dha: 2,3-Didehydroalanine
DhB: Didehydrobutyrine
LP: Leader peptide
CP: Core peptide
GFP: Green fluorescent protein
PTM: Post-translational modification
MCS: Multiple cloning site
RBS: Ribosomal binding site
GlyM: Glycerol presence in pre-growth media
GlyIn: Glycerol used as inducer agent
LB: Luria Bertani
TB: Terrific broth
TBG: TB containing glycerol
TB-G: TB without glycerol
BHI: Brain heart infusion media
IMAC: Immobilized metal affinity chromatography
hNisP: Heterologously produced nisin protease
STldh: Lactate dehydrogenase gene promoter of *Enterococcus mundtii* ST4SA
cAMP: Cyclic AMP
CAP: CAMP rceptor protein
LanD: Oxidoreductase
AviCys: *S*-[(Z)-2-aminovinyl]-d-cysteine
Amp: Ampicillin
Kan: Kanamycin
Cm: Chloramohenicol

## References

[1] Van Staden AD, Van Zyl WF, Trindade M, Dicks LMT, Smith C (2021). Therapeutic applications of lantibiotics and other lanthipeptides: old and new findings. Appl Environ Microbiol.

[2] Pei Z, Zhu L, Sarksian R, van der Donk WA, Nair SK (2022). Class V lanthipeptide cyclase directs the biosynthesis of a stapled peptide natural product. J Am Chem Soc.

[3] Arnison PG, Bibb MJ, Bierbaum G, Bowers AA, Bugni TS, Bulaj G (2013). Ribosomally synthesized and post-translationally modified peptide natural products: overview and recommendations for a universal nomenclature. Nat Prod Rep.

[4] Gross E, Morell JL (1967). The presence of dehydroalanine in the antibiotic nisin and its relationship to activity. J Am Chem Soc.

[5] Gross E, Morell JL, Craig LC (1969). Dehydroalanyllysine: identical COOH-terminal structures in the peptide antibiotics nisin and subtilin. Proc Natl Acad Sci USA.

[6] Gross E, Morell JL (1971). The structure of nisin. J Am Chem Soc.

[7] Barber M, Elliot GJ, Bordoli RS, Green BN, Bycroft BW (1988). Confirmation of the structure of nisin and its major degradation product by FAB-MS and FAB-MS/MS. Experientia.

[8] Yang X, van der Donk WA (2015). Michael-type cyclizations in lantibiotic biosynthesis are reversible. ACS Chem Biol.

[9] Bierbaum G, Szekat C, Josten M, Heidrich C, Kempter C, Jung G (1996). Engineering of a novel thioether bridge and role of modified residues in the lantibiotic Pep5. Appl Environ Microbiol.

[10] Kupke T, Kempter C, Gnau V, Jung G, Götz F (1994). Mass spectroscopic analysis of a novel enzymatic reaction. Oxidative decarboxylation of the lantibiotic precursor peptide EpiA catalyzed by the flavoprotein EpiD. J Biol Chem.

[11] Skaugen M, Nissen-Meyer J, Jung G, Stevanovic S, Sletten K, Inger C, Abildgaard M, Nes IF (1994). *In vivo* conversion of l-serine to d-alanine in a ribosomally synthesized polypeptide. J Biol Chem.

[12] Ökesli A, Cooper LE, Fogle EJ, van der Donk WA (2011). Nine post-translational modifications during the biosynthesis of cinnamycin. J Am Chem Soc.

[13] Oman TJ, van der Donk WA (2010). Follow the leader: the use of leader peptides to guide natural product biosynthesis. Nat Chem Biol.

[14] Rogers LA (1928). The inhibiting effect of *Streptococcus lactis* on *Lactobacillus bulgaricus*. J Bacteriol.

[15] Walker MC, Eslami SM, Hetrick KJ, Ackenhusen SE, Mitchell DA, van der Donk WA (2020). Precursor peptide-targeted mining of more than one hundred thousand genomes expands the lanthipeptide natural product family. BMC Genom.

[16] Ongey EL, Yassi H, Pflugmacher S, Neubauer P (2017). Pharmacological and pharmacokinetic properties of lanthipeptides undergoing clinical studies. Biotechnol Lett.

[17] Deng JJ, Viel JH, Kubyshkin V, Budisa N, Kuipers OP (2020). Conjugation of synthetic polyproline moietes to lipid II binding fragments of nisin yields active and stable antimicrobials. Front Microbiol.

[18] Montalbán-López M, Scott TA, Ramesh S, Rahman IR, van Heel AJ, Viel JH, Bandarian V, Dittmann E, Genilloud O, Goto Y (2021). New developments in RiPP discovery, enzymology and engineering. Nat Prod Rep.

[19] Ongey EL, Neubauer P (2016). Lanthipeptides: chemical synthesis versus *in vivo* biosynthesis as tools for pharmaceutical production. Microb Cell Fact.

[20] Mesa-Pereira B, Rea MC, Cotter PD, Hill C, Ross RP (2018). Heterologous expression of biopreservative bacteriocins with a view to low cost production. Front Microbiol.

[21] Herzner AM, Dischinger J, Szekat C, Josten M, Schmitz S, Yakéléba A, Reinartz R, Jansen A, Sahl HG, Piel J, Bierbaum G (2011). Expression of the lantibiotic mersacidin in *Bacillus amyloliquefaciens* FZB42. PLoS ONE.

[22] Krawczyk JM, Völler GH, Krawczyk B, Kretz J, Brönstrup M, Süssmuth RD (2013). Heterologous expression and engineering studies of labyrinthopeptins, class III lantibiotics from *Actinomadura namibiensis*. Chem Biol.

[23] van Heel AJ, Mu D, Montalbán-López M, Hendriks D, Kuipers OP (2013). Designing and producing modified, new-to-nature peptides with antimicrobial activity by use of a combination of various lantibiotic modification enzymes. ACS Synth Biol.

[24] Weixler D, Berghof M, Ovchinnikov KV, Reich S, Goldbeck O, Seibold GM, Wittmann C, Bar NS, Eikmanns BJ, Diep DB, Riedel CU (2022). Recombinant production of the lantibiotic nisin using *Corynebacterium glutamicum* in a two-step process. Microb Cell Fact.

[25] Boakes S, Appleyard AN, Cortés J, Dawson M (2010). Organization of the biosynthetic genes encoding deoxyactagardine B (DAB), a new lantibiotic produced by *Actinoplanes liguriae* NCIMB41362. J Antibiot.

[26] Meindl K, Schmiederer T, Schneider K, Reicke A, Butz D, Keller S (2010). Labyrinthopeptins: a new class of carbacyclic lantibiotics. Angew Chem Int Ed.

[27] Dabard J, Bridonneau C, Phillipe C, Anglade P, Molly D, Nardi M (2001). Ruminococcin A, a new lantibiotic produced by a *Ruminococcus gnavus* strain isolated from human feces. Appl Environ Microbiol.

[28] Shi Y, Yang X, Garg N, van der Donk WA (2011). Production of Lantipeptides in *Escherichia coli*. J Am Chem Soc.

[29] Zhang YI, Chen M, Bruner SD, Ding Y (2018). Heterologous production of microbial ribosomally synthesized and post-translationally modified peptides. Front Microb.

[30] Donovan RS, Robinson CW, Glick BR (1996). Review: Optimizing inducer and culture conditions for expression of foreign proteins under the control of the lac promoter. J Ind Microb.

[31] Rollema HS, Kuipers OP, Both P, de Vos WM, Siezen RJ (1995). Improvement of solubility and stability of the antimicrobial peptide nisin by protein engineering. Appl Environ Microbiol.

[32] Wilson-Stanford S, Kalli A, Håkansson K, Kastrantas J, Orugunty RS, Smith L (2009). Oxidation of lanthionines renders the lantibiotic nisin inactive. Appl Environ Microbiol.

[33] Van staden AD. *In vitro* and *in vivo* characterization of amyloliquecidin, a novel two-component lantibiotic produced by *Bacillus amyloliquefaciens*. Thesis (PhD). Stellenbosch University. 2015.

[34] Ortega MA, Hao Y, Walker MC, Donadio S, Sosio M, Nair SK, van der Donk WA (2016). Structure and tRNA specificity of MibB, a lantibiotic dehydratase from actinobacteria involved in NAI-107 biosynthesis. Cell Chem Biol.

[35] Bothwell IR, Cogan DP, Kim T, Reinhardt CJ, van der Donk WA, Nair SK (2019). Characterization of glutamyl-tRNA-depended dehydratases using nonreactive substrate mimics. Proc Natl Acad Sci USA.

[36] Li B, Cooper LE, van der Donk WA, Hopwood D (2009). *In vitro* studies of lantibiotic biosynthesis. Methods in enzymology.

[37] Van Staden AD, Faure LM, Vermeulen RR, Dicks LMT, Smith C (2019). Functional expression of GFP-fused class I lanthipeptides in *Escherichia coli*. ACS Synth Biol.

[38] Ongey EL, Giessmann RT, Fons M, Rappsilber J, Adrian L, Neubauer P (2018). Heterologous biosynthesis, modifications and structural characterization of ruminococcin-A, a lanthipeptide from the gut bacterium *Ruminococcus gnavus* E1, *in Escherichia coli*. Front Microbiol.

[39] Si T, Tian Q, Min Y, Zhang L, Sweedler JV, van der Donk WA, Zhao H (2018). Rapid screening of lanthipeptide analogs via in-colony removal of leader peptides in *Escherichia coli*. J Am Chem Soc.

[40] Van Zyl WF, Deane SM, Dicks LMT (2018). *In vivo* bioluminescence imaging of the spatial and temporal colonization of *Lactobacillus plantarum* 423 and *Enterococcus mundtii* ST4SA in the intestinal tract of mice. BMC Microbiol.

[41] Andersen HW, Pedersen MB, Hammer K, Jensen PR (2001). Lactate dehydrogenase has no control on lactate production but has a strong negative control on formate production in *Lactococcus lactis*. Eur J Biochem.

[42] Van Zyl WF, Deane SM, Dicks LMT (2019). Bacteriocin production and adhesion properties as mechanisms for the anti-listerial activity of *Lactobacillus plantarum* 423 and *Enterococcus mundtii* ST4SA. Benef Microbes.

[43] Cotter PD, Ross RP, Hill C (2013). Bacteriocins—a viable alternative to antibiotics?. Nat Rev Microbiol.

[44] Dischinger J, Chipalu SB, Bierbaum G (2013). Lantibiotics: promising candidates for future applications in health car. Int J Med Microb.

[45] Vermeulen RR, Van Staden AD, Dicks L (2020). Heterologous expression of the class IIa bacteriocins, plantaricin 423 and mundticin ST4SA, in *Escherichia coli* using green fluorescent protein as a fusion partner. Front Microbiol.

[46] Kuthning A, Mosker E, Sussmuth RD (2015). Engineering the heterologousexpression of lanthipeptides in *Escherichia coli* by multigene assembly. Appl Microbiol Biotechnol.

[47] Chapagain PP, Regmi CK, Castillo W (2011). Fluorescent protein barrel fluctuations and oxygen diffusion pathways in mCherry. J Chem Phys.

[48] Doherty GP, Bailey K, Lewis PJ (2010). Stage-specific fluorescence intensity of GFP and mCherry during sporulation in *Bacillus subtilis*. BMC Res Notes.

[49] Shearwin KE, Callen BP, Egan JB (2005). Transcriptional interference—a crash course. Trends Genet.

[50] Palmer AC, Egan JB, Shearwin KE (2011). Transcriptional interference by RNA polymerase and dislodgement of transcriptional factors. Transcription.

[51] Toyoda K, Inui M (2021). The *ldhA* gene encoding fermentative l-lactate dehydrogenase in *Corynebaterium glutamicum* is positively regulated by the global regulator GlxR. Microorganisms.

[52] Grossman TH, Kawasaki ES, Punreddy SR, Osburne MS (1998). Spontaneous cAMP-dependent derepression of gene expression in stationary phase plays a role in recombinant expression instability. Gene.

[53] Bentenbaugh MJ, Dhurjatl P (1990). A comparison of mathematical model predictions to experimental measurements for growth and recombinant protein production in induced cultures of *Escherichia coli*. Biotechnol Bioeng.

[54] DeBellis D, Schwartz I (1990). Regulated expression of foreign genes fused to lac: control by glucose levels in the growth medium. Nucl Acids Res.

[55] Wurm DJ, Hausjell J, Ulonska S, Herwig C, Spadiut O (2017). Mechanistic platform knowledge of concomitant sugar uptake in *Escherichia coli* BL21(DE3) strains. Sci Rep.

[56] Martínez-Gómez K, Flores N, Castañeda HM, Martínez-Batallar G, Hernández-Chávez G, Ramírez OT, Gosset G, Encarnación S, Bolivar F (2012). New insights into *Escherichia coli* metabolism: carbon scavenging, acetate metabolism and carbon recycling responses during growth on glycerol. Microb Cell Fact.

[57] Lin EC (1976). Glycerol dissimilation and its regulation in bacteria. Annu Rev Microbiol.

[58] Kopp J, Slouka C, Ulonska S, Kager J, Fricke J, Spadiut O, Herwig C (2021). Impact of glycerol as carbon source onto specific sugar and inducer uptake rates and inclusion body productivity in *E. coli* BL21 (De3). Bioengineering.

[59] Glick BR (1995). Metabolic load and heterologous gene expression. Biotechnol Adv.

[60] Rosano GL, Ceccarelli EA (2014). Recombinant protein expression in microbial systems. Front Microbiol.

[61] Pal G, Srivastava S (2015). Scaling up the production of recombinant antimicrobial plantaricin E from a Heterologous Host, *Escherichia coli*. Probiotics Antimicrob Prot.

[62] Rogne P, Fimland G, Nissen-Meyer J, Kristiansen PE (2008). Three-dimensional structure of the two peptides that constitute the twopeptide bacteriocin lactococcin G. Biochim Biophys Acta.

[63] Masias E, Picariello G, Acuña L, Chalon M, Sesma F, Morero R (2014). Co-expression and characterization of enterocin CRL35 and its mutant in *Escherichia coli* Rosetta. Peptidomics.

[64] Glassey E, King AM, Anderson DA, Zhang Z, Voight CA (2022). Functional expression of diverse post-translational peptide-modifying enzymes in *Escherichia coli* under uniform expression and purification conditions. PLoS ONE.

[65] Fuller F (1982). A family of cloning vectors containing the lac UV5 promoter. Gene.

[66] Solaiman DKY, Somkuti GA (1991). Expression of streptomycete cholesterol oxidase in *Escherichia coli*. J Ind Microbiol.

[67] Schnell N, Engelke G, Augustin J, Rosenstein R, Ungermann V, Gotz F, Entian KD (1992). Analysis of genes involved in the biosynthesis of lantibiotic epidermin. Eur J Biochem.

[68] Sit CS, Yoganathan S, Vederas JC (2011). Biosynthesis of aminovinyl-cysteine containing peptides and its application in the production of potential drug candidates. Accounts Chem Res.

[69] Schnell N, Entian K, Schneider U, Gotz F, Zahner H, Kellner R, Jung G (1988). Prepeptide sequence of epidermin, a ribosomaly synthesized antibiotic with four sulphide-rings. Lett Nature.

[70] Van Staden AD, Heunis T, Smith C, Deane S, Dicks LMT (2016). Efficacy of lantibiotic treatment of *Staphylococcus aureus*-induced skin infections, monitored by *in vivo* bioluminescent imaging. Antmicrob Agents Chemother.

[71] Urdaci MC, Bressollier P, Pinchuk I (2004). *Bacillus clausii* probiotic strains: antimicrobial and immunomodulatory activities. J Clin Gastroenterol.

[72] Bouhss A, Al-Dabbagh B, Vincent M, Odaert B, Aumont-Nicaise M, Bressolier P, Desmadril M, Mengin-Lecreulx D, Urdaci MC, Gallay J (2009). Specific interactions of clausin, a new lantibiotic, with lipid precursors of the bacterial cell wall. Biophys J.

[73] Augustin J, Rosenstein R, Wieland B, Schneider U, Schnell N, Engelke G, Entian KD, Gotz F (1992). Genetic-analysis of epidermin biosynthetic genes and epidermin-negative mutants of *Staphylococcus epidermidis*. Eur J Biochem.

[74] Ausubel FM, Brent R, Kingston RE, Moore DD, Seidman JG, Smith JA, Struhl K (1994). Current protocols in molecular biology.

[75] Sambrook J, Russell D (2001). Molecular cloning: a laboratory manual.

