# Supplementary material for: Use of the mCherry fluorescent protein to optimize the expression of class I lanthipeptides in Escherichia coli
Source: Microb Cell Fact. 2023 Aug 9;22:149. doi: 10.1186/s12934-023-02162-7 (PMC10413542; doi:10.1186/s12934-023-02162-7)
Supplement: Supplementary file 1 — Additional file 1: Figure S1. Growth curves of E. coli BL21 strains NisSTldhSTldh and NisSTldhT7 expressing mCherry-NisA. Each vector with the respective promoter combinations was co-transformed with pACYCNisCi. The negative non-fluorescent control expression strain (Control) was transformed with empty pRSF-Duet 1 and pACYCNisCi. STldhSTldh = STldh promoter used for expression of mCherry-NisA and NisB; STldhT7 = STldh used for expression of mCherry-NisA and T7 promoter for NisB. Figure S2. Growth profiles of strain NisSTldhT7 expressing mCherry-NisA under various culturing and induction conditions. Cultures were grown in terrific broth supplemented without (A) and with (B) glucose (1% v/v) for 24 h. Control cultures were transformed with empty pRSF Duet-1 (without mCherry) and pACYC Duet-1 plasmids, while recombinant E. coli BL21 were transformed with pRSTldhCherryPrenisinNisB and pACYC-NisC plasmids. Culturing and induction conditions included: (−IPTG; −GlyM; −GlyIn) no induction; (−IPTG; +GlyM; −GlyIn) no IPTG induction with glycerol (0.5% v/v) added to pre-growth media; (−IPTG; −GlyM; +GlyIn) no IPTG induction but induced with glycerol (0.5% v/v); (+IPTG; +GlyM; −GlyIn) induced with 0.15 mM IPTG and glycerol added to pre-growth media; (+IPTG; −GlyM; +GlyIn) induced with 0.15 mM IPTG and glycerol; (+IPTG; −GlyM; −GlyIn) induced with 0.15 mM IPTG. IPTG = Thio-B-d-galactopyranoside; GlyM = glycerol presence in pre-growth media; GlyIn = glycerol used as inducer agent; +: added and −: not added. Figure S3. Growth profiles of strain NisSTldhT7 expressing mCherry-NisA in M9 minimal media, LB, BHI, TBG (TB containing 0.5% glycerol), TB-G (TB without 0.5% glycerol), A induced with 0.15 mM IPTG and 0.5% glycerol (v/v) and B un-induced. Figure S4. A pRSTc*nB backbone plasmid map. B Close up view of gene fragments. Relevant plasmid elements, promoters, genes and restriction sites are shown. Lanthipeptide genes are inserted at PstI/NotI restriction enzyme sites. Figure S5. A [file 12934_2023_2162_MOESM1_ESM.docx]

**Additional file 1**

**Use of the mCherry Fluorescent Protein to Optimize the Expression of Class I Lanthipeptides in *Escherichia coli***

Winschau F. van Zyl^1, 4*^, Anton D. van Staden^1,3^, Leon MT. Dicks^1^ and Marla Trindade^2^

^1^ Department of Microbiology, Stellenbosch University, Cape Town, South Africa

^2^ Institute for Microbial Biotechnology and Metagenomics, University of the Western Cape, Cape Town, South Africa

^3^ Division of Clinical Pharmacology, Department of Medicine, Faculty of Medicine and Health Sciences, Stellenbosch University, Cape Town, South Africa

^4^ Department of Microbiology and Biochemistry, University of the Free State, Bloemfontein, South Africa

* Corresponding Author: Winschau F. Van Zyl ([vanzylwf1@ufs.ac.za](mailto:vanzylwf@ufs.ac.za))

**Fig. S1** Growth curves of *E. coli* BL21 strains NisSTldhSTldh and NisSTldhT7 expressing mCherry-PreNisA. Each vector with the respective promoter combinations were co-transformed with pACYCNisCi. The negative non-fluorescent control expression strain (Control) was transformed with empty pRSF-Duet 1 and pACYCNisCi. STldhSTldh = STldh promoter used for expression of mCherry-NisA and NisB; STldhT7 = STldh used for expression of mCherry-NisA and T7 promoter for NisB.

S2. B

S2. A

**Fig. S2** Growth profiles of strain NisSTldhT7 expressing mCherry-PreNisA under various culturing and induction conditions. Cultures were grown in terrific broth supplemented without **(A)** and with **(B)** glucose (1% v/v) for 24 h. Control cultures were transformed with empty pRSF Duet-1 (without mCherry) and pACYC Duet-1 plasmids, while recombinant *E. coli* BL21 were transformed with pRSTldhCherryPrenisinNisB and pACYC-NisC plasmids. Culturing and induction conditions included: (-IPTG; -GlyM; -GlyIn) no induction; (-IPTG; +GlyM; -GlyIn) no IPTG induction with glycerol (0.5% v/v) added to pre-growth media; (-IPTG; -GlyM; +GlyIn) no IPTG induction but induced with glycerol (0.5% v/v); (+IPTG; +GlyM; -GlyIn) induced with 0.15 mM IPTG and glycerol added to pre-growth media; (+IPTG; -GlyM; +GlyIn) induced with 0.15 mM IPTG and glycerol; (+IPTG; -GlyM; -GlyIn) induced with 0.15 mM IPTG. IPTG = Thio-B-D-galactopyranoside; GlyM = glycerol presence in pre-growth media; GlyIn = glycerol used as inducer agent; + = added and - = not added.

S3. B

S3. A

**Fig. S3** Growth profiles of strain NisSTldhT7 expressing mCherry-PreNisA in M9 minimal media, LB, BHI, TBG (TB containing 0.5% glycerol), TB-G (TB without 0.5% glycerol), **(A)** induced with 0.15 mM IPTG and 0.5% glycerol (v/v) and **(B)** un-induced.


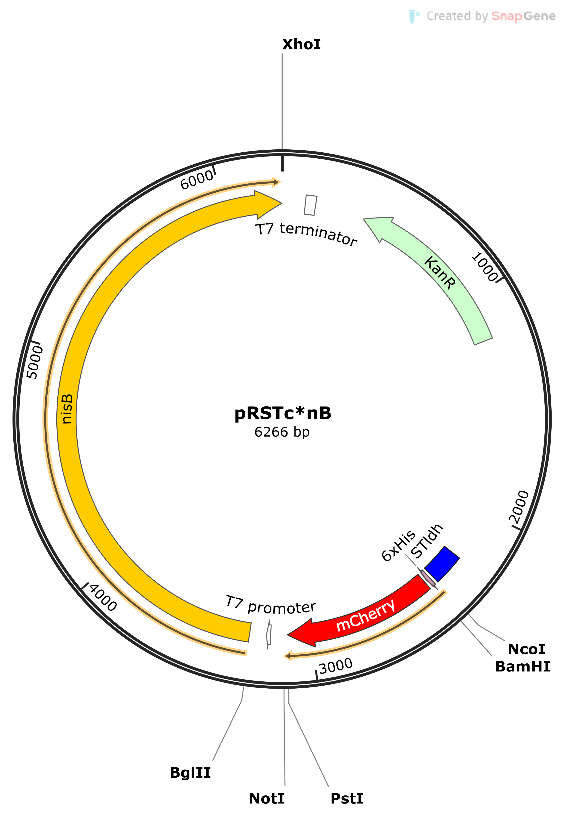


S4. A

S4. B


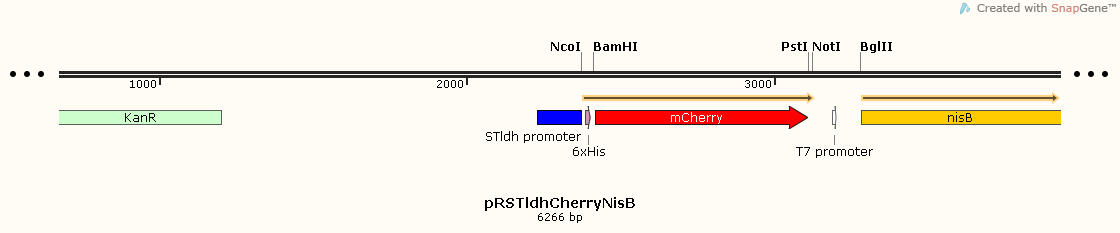


**Fig. S4 (A)** pRST*c*nB* backbone plasmid map. **(B).** Close up view of gene fragments. Relevant plasmid elements, promoters, genes and restriction sites are shown. Lanthipeptide genes are inserted at PstI/NotI restriction enzyme sites.


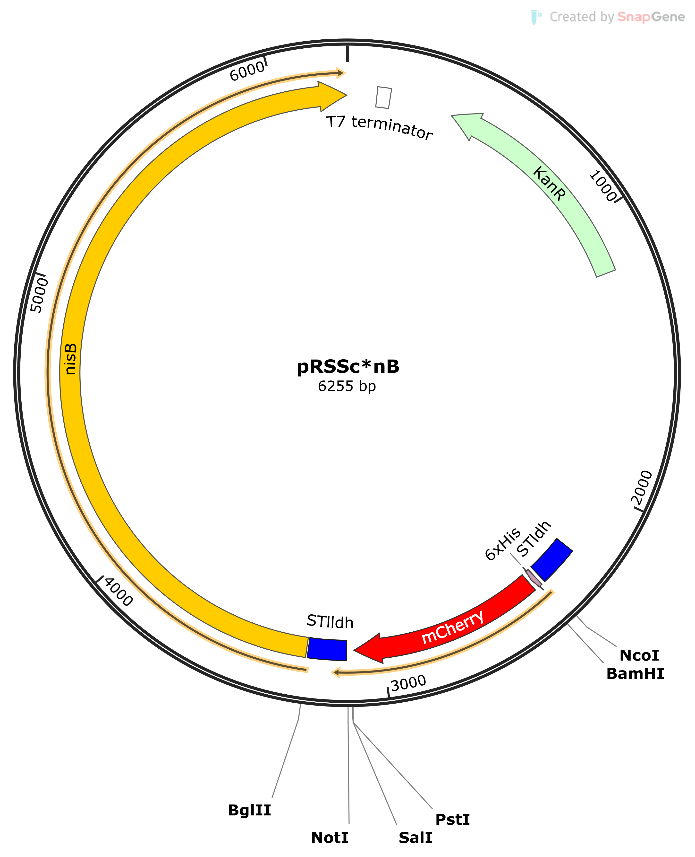


S5. A

S5. B


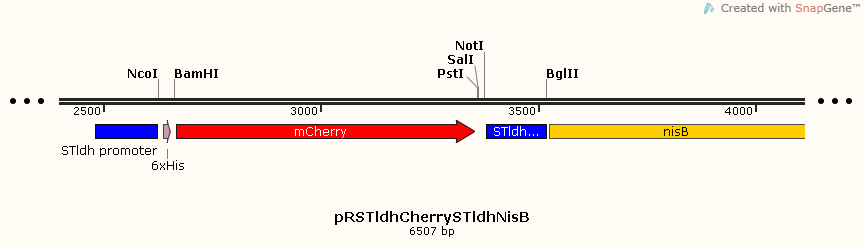


**Fig. S5 (A)** pRSS*c*nB* backbone plasmid map. **(B).** Close up view of gene fragments. Relevant plasmid elements, promoters, genes and restriction sites are shown. Lanthipeptide genes are inserted at PstI/NotI restriction enzyme sites. The T7 promoter in MCS2 was replaced with a second STldh promoter using NotI/BglII restriction enzyme sites.


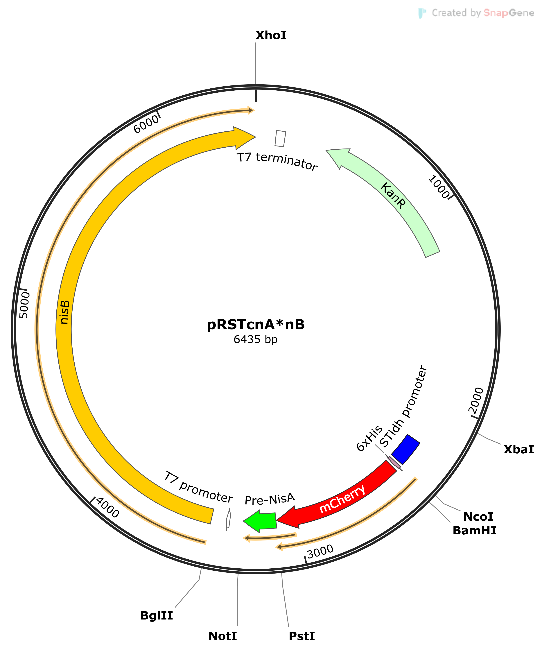

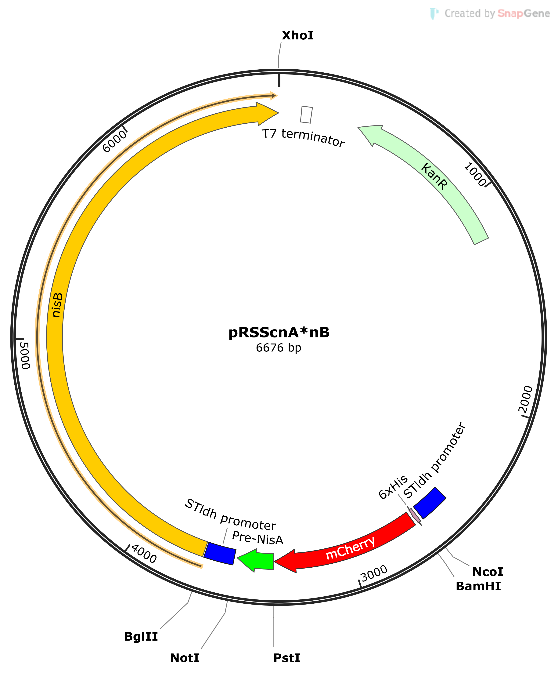


S6. B

S6. A


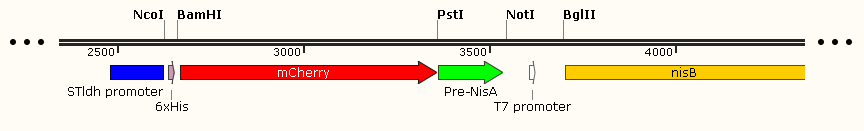


S6. C

S6. D


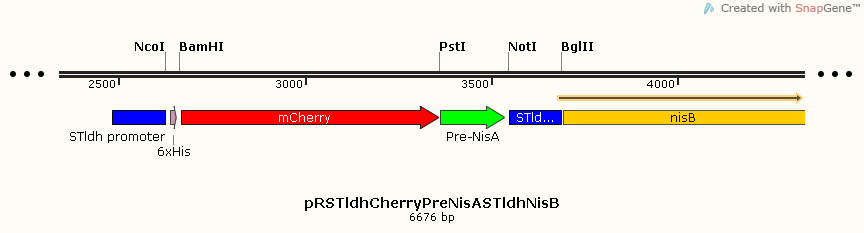


**Fig. S6 (A)** pRST*cnA*nB* and **(B)** pRSS*cnA*nB* mCherry-nisA heterologous expression plasmids. **(C and D).** Linearized views of gene fragments shown in A and B, respectively. Relevant plasmid elements, promoters, genes and restriction sites are shown.


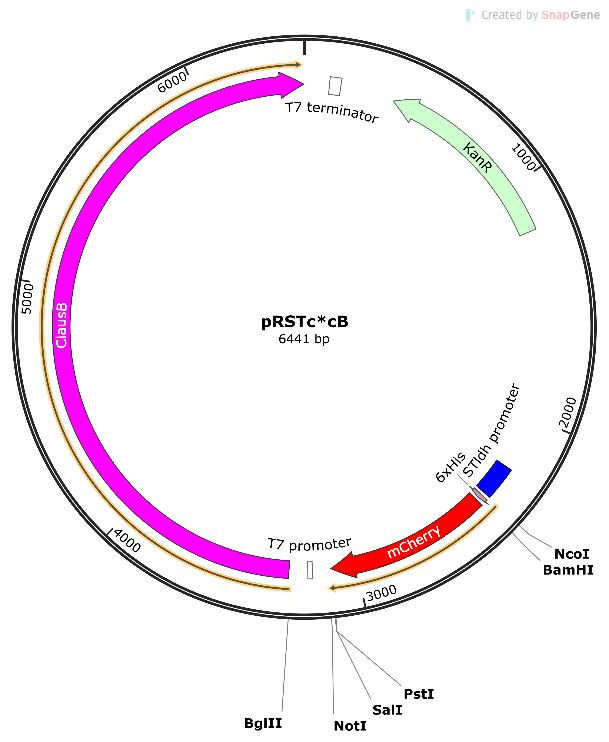


S7. A

S7. B


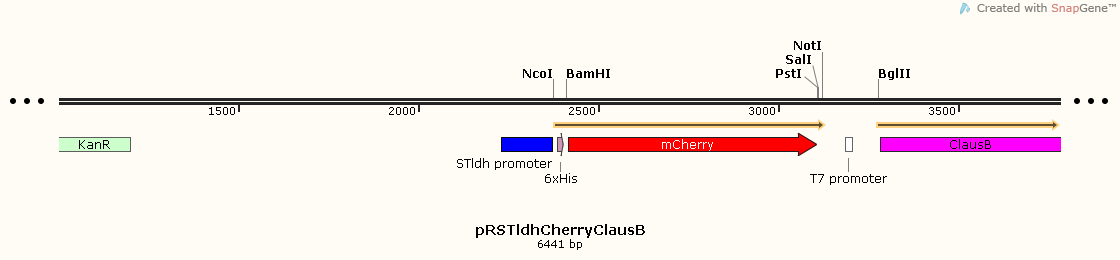


**Fig. S7 (A)** pRST*c*cB* backbone plasmid map. **(B).** Close up view of gene fragments. Relevant plasmid elements, promoters, genes and restriction sites are shown. Lanthipeptide genes are inserted at PstI/NotI restriction enzyme sites.


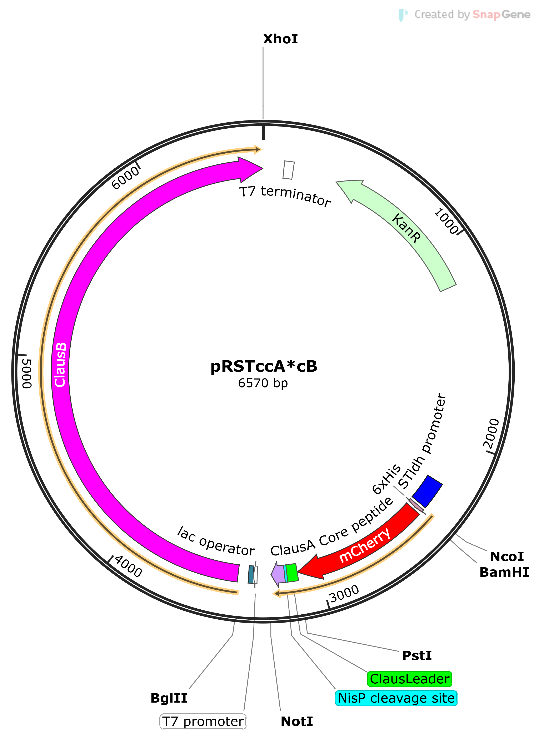

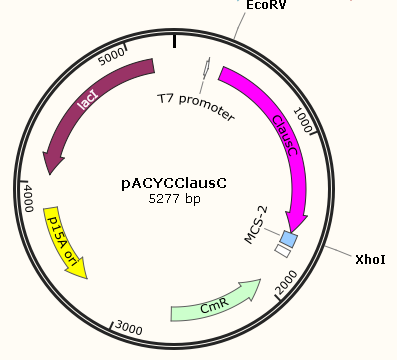

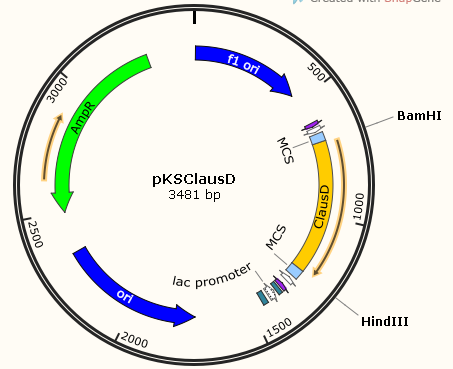


S8. D

S8. C

S8. B

S8. A


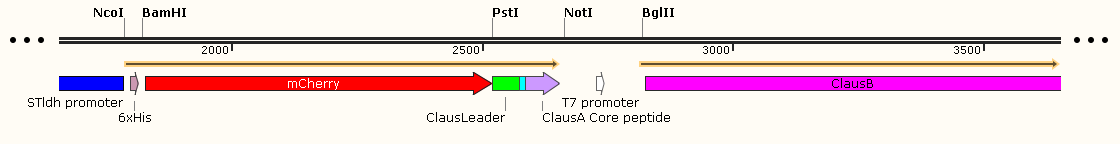


NisP cleavage site


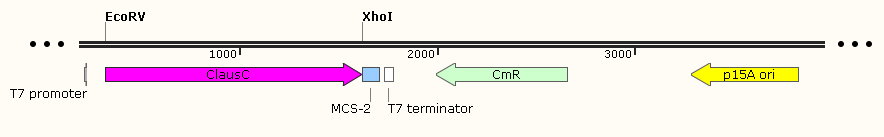


S8. E


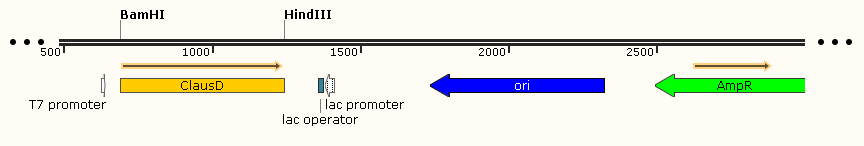


S8. F

**Fig. S8 (A)** pRST*ccA*cB* mCherry-ClausA heterologous expression plasmid, **(B)** pClausCi clausC cyclase expression plasmid and **(C)** pClausDi clausD expression plasmid. **(D, E and F).** Close up and linearized views of gene fragments shown in A, B and C, respectively. Relevant plasmid elements, promoters, genes and restriction sites are shown.


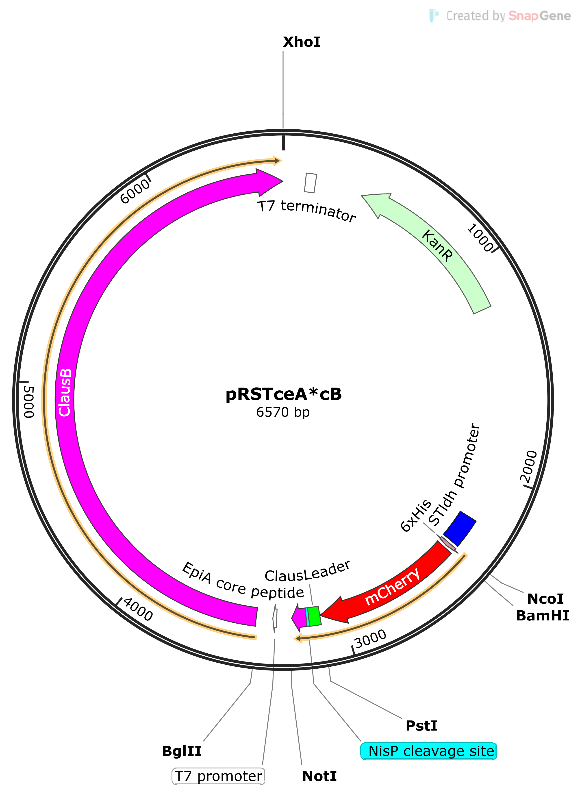


S9. B

S9. A


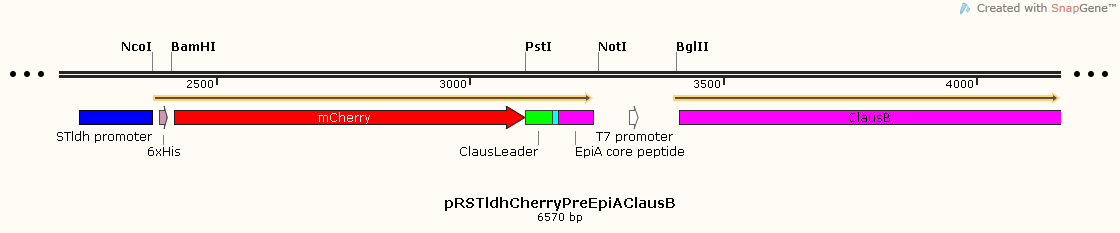


NisP cleavage site

**Fig. S9 (A)** pRST*ceA*cB* backbone plasmid map. **(B).** Close up view of gene fragments. Relevant plasmid elements, promoters, genes and restriction sites are shown. Lanthipeptide genes are inserted at PstI/NotI restriction enzyme sites.

**1** **2**


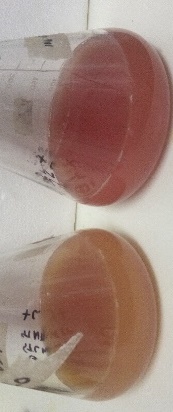


**Fig. S10** Differentiation of mCherry-PreNisA production based on color produced by host cells in spent expression media carrying the promoter arrangement described for strains **(1)** NisSTldhSTldh and **(2)** NisSTldhT7, respectively, after 20 h incubation.


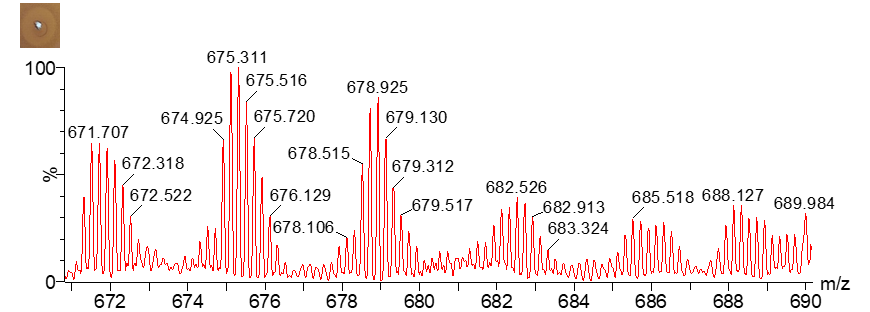


**Fig. S11** Mass spectrum of hNisP cleaved mCherry-PreNisA produced in *E. coli*. Activity zones are representative of cleaved and extracted samples before ESI-MS analysis.

**Off On**

M9
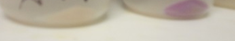

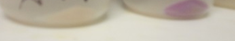


LB
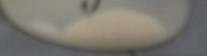

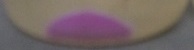


BHI
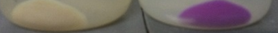

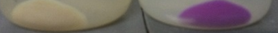


TBG
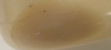

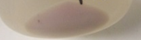


TB-G
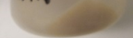

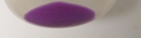


**Fig. S12** Visual representation of colour differentiation of cell pellets produced when cultures are kept uninduced (off) and when induced (on).

**Table S1** Bacterial strains and plasmids used in this study

| **Strain or plasmid** | **Description** | **Reference or source** |
| --- | --- | --- |
| **Strain** |  |  |
| *E. coli* |  |  |
| DH5α | Host strain used for general subcloning | (1) |
| BL21 (DE3) | Expression host strain | Lab culture collection |
| *Lactococcus lactis* QU2 | Nisin A producer | Gift from Prof. K. Sonomoto |
| *Enterococcus mundtii* ST4SA | Source strain of STldh promoter | Culture collection, Department of Microbiology, Stellenbosch University |
| *Bacillus clausii* AD1 | Clausin producer | (2) |
| *Lactobacillus sakei* 20017  **Plasmid** | Lanthipeptide sensitive organisms | Culture collection, Department of Microbiology, Stellenbosch University |
| pRSFDuet-1 | High copy number expression vector carrying the IPTG inducible T7 promoter in MCS1 and MCS2; Cloning site for N-terminal 6x His tag fusion in MCS1; T7 transcriptional terminator; Kan^R^ | Novagen |
| pACYCDuet-1 | Low copy number expression vector carrying the IPTG inducible T7 promoter in MCS1 and MCS2; Cloning site for N-terminal 6x His tag fusion in MCS1; Cm^R^ | Novagen |
| pNZSTldhMCS1-2 | Source vector carrying the constitutive *E. mundtii* ST4SA lactate dehydrogenase gene STldh promoter and two multiple cloning sites (MCS); Cm^R^ | Unpublished study |
| pRSTldhMCS1-2 | pRSFDuet-1 vector carrying the STldh constitutive promoter upstream of MCS 1; Cloning site for N-terminal 6x His tag fusion in MCS1; T7 promoter in MCS 2; Kan^R^ | This study |
|  |  |  |
| pRSFGFP | GFP source vector; pRSFDuet-1 vector carrying GFP with an N-terminal 6x His Tag and C-terminal WELQut site in MCS1; Kan^R^ | (3) |
| pNZSTldhCherry | mCherry source vector carrying mCherry under control of the constitutive *E. mundtii* ST4SA lactate dehydrogenase gene STldh promoter; Cm^R^ | Unpublished study |
| pRSTldhGFP | pRSTldhMCS1-2 vector carrying mCherry with a C-terminal WELQut site in MCS1; Kan^R^ | This study |
| pRSTldhCherry | pRSTldhMCS1-2 vector carrying mCherry with a C-terminal WELQut site in MCS1; Kan^R^ | This study |
| pRST*c*nB* | pRSTldhCherry vector carrying *nisB* NisinA dehydratase in MCS2; Kan^R^ | This study |
| pRST*cnA*nB* | pRST*c*nB* vector carrying Pre-NisA (original ASPR hNisP cleavage site for removal of Nisin leader) fused to mCherry; Kan^R^ | This study |
| pRSS*c*nB* | pRST*c*nB* vector carrying the constitutive *E. mundtii* ST4SA lactate dehydrogenase gene STldh promoter in MCS’s 1 and MCS2; Kan^R^ | This study |
| pRSS*cnA*nB* | pRSS*c*nB* carrying Pre-NisA (original ASPR hNisP cleavage site for removal of Nisin leader) fused to mCherry; Kan^R^ | This study |
| pRST*c*cB* | pRSTldhCherry vector carrying the clausB Clausin dehydratase in MCS2; Kan^R^ | This study |
| pRST*ccA*cB* | pRST*c*cB* vector carrying the clausin leader-Clausin core peptide fused to mCherry (ASPR, hNisP cleavage site); Kan^R^ | This study |
| pRST*ceA*cB* | pRST*c*cB* vector carrying the clausin leader-EpiA core peptide fused to mCherry (ASPR, hNisP cleavage site); Kan^R^ | This study |
| pACYCNisCi | pACYCDuet-1 vector carrying the *nisC* NisinA cyclase for co-expression of NisC in MCS2; Cm^R^ | (3) |
| pClausCi | pACYCDuet-1 vector carrying | This study |
|  | *clausC* for Clausin cyclase for co-expression of ClausC in MCS2; Cm^R^ |  |
| pBluescriptKS | High-copy number PCR cloning vector carrying the IPTG inducible T7 promoter and a MCS; Amp^R^ | This study |
| pClausDi | pBluescriptKS plasmid carrying *clausD* for Clausin amino-vinyl cysteine formation in the MCS; Amp^R^ | This study |
| pRSF-hNisPi | pRSFDuet-1 vector carrying truncated NisP with a C-terminal 8x His tag; Kan^R^ | (3) |

Cm^R^, chloramphenicol resistance; Kan^R^, kanamycin resistance; Amp^R^, ampicillin resistance; IPTG, Thio-B-D galactopyranoside; MCS, multiple cloning site; STldh, constitutive lactate dehydrogenase gene promoter.

**Table S2. Oligonucleotides utilized in this study**

| **Target** | **Primer** | **Sequence 5’ to 3’** | **Restriction sites** | **Product size (bp)** |
| --- | --- | --- | --- | --- |
| *nisB* dehydratase | nisB1 | CAGCCG**AGATCT**GATGATAAAAAGTTCATTTAAAGCTCAACCG | *Bgl*II | 2982 |
|  | nisB2 | CTAG**CTCGAG**TCATTTCATGTATTCTTCCGAAACAAACAACC | *Xho*I |  |
| STldh promoter | STldh1 | GCTTGGAT**CCATGG**TAAGCCGATACGTACC | *Not*I | 156 |
|  | STldh2 | AGTC**AAGCTT**CTACCCAATCAGTACGTTAATT | *Bgl*II |  |
| *clausB* dehydratase | clausB1 | GCGC**AGATCT**GATGGCGATGAGAAAAAGC | *Bgl*II | 3156 |
|  | ClausB2 | GCGC**CTCGAG**TCATACGTTTAATGGTTG | *Xho*I |  |
| *clausC* dehydratase | ClausC1 | GCGC**GATATC**gATGATTGCCCATTTGA | *Eco*RV | 1299 |
|  | ClausC2 | GCGC**CTCGAG**CTAACTGATTAAAAACGC | *Xho*I |  |
| *clausD* | clausD1 | GCGC**CCATGG**GATGCCCTCCTATTTAG | *Nco*I | 510 |
|  | clausD2 | GCGC**AAGCTT**CTAGCTTTTTTCTTTTAC |  |  |
| *nisA* | nisA1 | CTAGAT**GGATCC**GATGAGTACAAAAGATTTTAACTTGG | *BamHI* | 174 |
|  | nisA2 | CTAG**AAGCTT**TTATTTGCTTACGTGAATACTACAATG | *HindIII* |  |
| *epiA* | epiAFor | CATG**CTGCAG**GATGGAAGCAGTAAAA | *Pst*I | 159 |
|  | EpiANotIRev | CATG**GCGGCCGC**TTAACAACAATAACTGTTAA | *Not*I |  |
| ClausA core with 5’ hNisP sequence overlap | ClausNisPLanOL | GCATCACCACGCTTCACAAGCGTCAGTTT | - | 89 |
| epiA core with 5’ hNisP sequence overlap | EpiANisPOL | GCATCACCACGCATTGCTAGTAAATTTATATGTA | *-* | 94 |
| *clausA* leader fusion to clausA/epiA core peptides | ClausLFor | CTCA**CTGCAG**GATGGAAAAAGCTTTTGA | *Pst*I | 76 |
|  | ClausLNisPRevOL | GCGTGGTGATGCATCCTTCGCTTTCGTGT | - |  |
|  | ClausANotIRev | ATAATTTA**GCGGCCGC**TTAGCAGCAGAAGCTATTAAAGC | *Not*I | 135 |

**References**

1. Ausubel FM, Brent R, Kingston RE, Moore DD, Seidman JG, Smith JA, Struhl K. Current protocols in molecular biology. New York: Wiley. 1994.
2. Van Staden AD, Heunis T, Smith C, Deane S, Dicks LMT. Efficacy of lantibiotic treatment of Staphylococcus aureus-induced skin infections, monitored by in vivo bioluminescent imaging. Antmicrob Agents Chemother.2016;60(7):3948-55.
3. Van Staden AD, Faure LM, Vermeulen RR, Dicks LMT, Smith C. Functional expression of GFP-fused class I lanthipeptides in *Escherichia coli.* ACS Synth Biol. 2019;8:2220–7.
